# Some bats are here: Reducing the Wallacean shortfall of bats in the amazon

**DOI:** 10.1002/ece3.11392

**Published:** 2024-06-04

**Authors:** Thiago Bernardi Vieira, Rafaela Jemely Rodrigues Alexandre, Simone Almeida Pena, Letícia Lima Correia, Ariane de Sousa Brasil, Ludmilla Moura de Souza Aguiar, Paulo De Marco, Albert David Ditchfield

**Affiliations:** ^1^ Laboratório de Ecologia, Faculdade de Ciências Biológicas (FCB) Universidade Federal do Pará (UFPA) Altamira PA Brazil; ^2^ Programa de Pós‐graduação em Zoologia (PPGZOOL) Universidade Federal do Pará Belém Brazil; ^3^ Programa de Pós‐Graduação em Ecologia (PPGECO) Universidade Federal do Pará Belém Brazil; ^4^ Programa de Pós‐Graduação em Ecologia, Laboratório de Biologia e Conservação de Morcegos, Departamento de Zoologia, Instituto de Ciências Biológicas Universidade de Brasília Brasília DF Brazil; ^5^ Theoretical, Metacommunity and Landscape Ecology Laboratory, Instituto de Ciências Biológicas Universidade Federal de Goiás Goiás Brazil; ^6^ Laboratório de Estudos em Quirópteros (LABEQ), Departamento de Ciências Biológicas Universidade Federal do Espírito Santo (UFES) Vitória ES Brazil

**Keywords:** Amazonian Forest, Chiroptera, gap analyses, Phyllostomidae, SDM, Wallacean shortfall

## Abstract

The Amazon rainforest has approximately 23% of its sampled area dedicated to bats, making it one of the least sampled and most diverse regions for bats in Brazil. The lack of sampling results in a lack of knowledge regarding the accurate geographical distribution of bat species. This lack is referred to as the Wallacean shortfall, which should be addressed with primary data obtained from in situ collections. However, the use of Species Distribution Models (SDMs) can help alleviate this gap. The states of Pará and Acre are located in the Brazilian Amazon. So, our objective is to decrease the Wallacean shortfall concerning Amazonian bat species. To achieve this, we provide (i) a list of bat species sampled in the states of Pará and Acre in the last 5 years (2017 to 2022); (ii) the potential distribution of species considered as new occurrences for the region; and (iii) the potential distribution of species classified as Data Deficient (DD) and Near Threatened (NT) according to the IUCN classification. With 96 nights of collection and 129,600 m^2^h of mist netting, we obtained 75 bat species, with an estimated total of 94.78 species. Additionally, 21 species were considered as range extensions. The Brazilian Amazon region has a vast geographic expanse and few established research centers, resulting in a limited sampling of bats and other biological groups. Furthermore, we draw attention to the significant number of bat species with expanded geographical distributions, with 21 out of the 75 sampled species. This should be a reminder that primary biogeographic data is still necessary for the neotropical region.

## Graphical abstract

New occurrences of bats in the Brazilian Amazon.

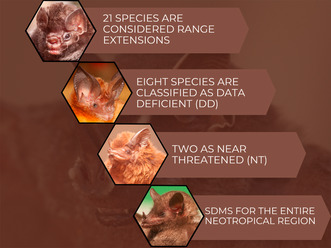

## 1 INTRODUCTION

The gap in knowledge about the geographical distribution of species, referred to as the Wallacean shortfall, is one of the challenges encountered in implementing conservation strategies effectively (Diniz‐Filho et al., 2013; Nóbrega & De Marco Jr, 2011; Sousa‐Baena et al., 2014). This knowledge gap about species distribution reflects the scarcity or complete absence of primary biogeographical data. Despite research involving the collection of biogeographical data having been conducted for more than two centuries (Figueiró, 2015), many areas still remain under‐sampled or unsampled altogether (Lobo et al., 2018; Lomolino, 2004), and this is relatively common in the Neotropical region.

In the Amazon rainforest, considered the largest and most diverse expanse of tropical forest on the planet (López‐Baucells et al., 2019; Smith et al., 2023), approximately 23% of its area is sampled for bats, housing one in every ten known bat species (Bernard et al., 2012), in contrast to the 85% in the Atlantic Forest, the Brazilian biome best‐sampled for bats (Aguiar et al., 2020; Bernard et al., 2012). However, we observe rapid land use and cover changes driven by deforestation and wildfires (Gomez et al., 2015; Silva et al., 2016). This swift change in land use and land cover, coupled with the lack of knowledge about species distribution, results in ineffective conservation strategies since many of the areas classified as mega‐diverse, both in terms of species and ecosystem services, tend to overlap with deforested areas (Brasileiro et al., 2022; Delgado‐Jaramillo et al., 2020). Approximately 16.05% of the Amazon biome is no longer suitable for the ecosystem services bats provide (Brasileiro et al., 2022). This loss reflects 17% of the total area of the biome that has been deforested and converted into pastures and short‐cycle crops (Projeto MapBiomas, 2023).

This degradation of natural areas has a negative impact both on the climate as the forest is now considered a source of carbon emissions (Gatti et al., 2021), and on bats (Estrada & Coates‐Estrada, 2002; Palheta et al., 2020; Vieira et al., 2021), leading to local extinctions in certain areas of the anthropocene (Hutson et al., 2001; Voigt & Kingston, 2016) and long‐term effects as in the Brazilian cerrado, for example (dos Santos et al., 2016). Bats are considered keystone species in neotropical environments (Kunz & Fenton, 2005), playing crucial roles as seed dispersers (Kasso & Balakrishnan, 2013; Suripto, 2021), essential agents in reforestation of degraded environments (Muscarella & Fleming, 2007), and in pollination (Baqi et al., 2022; Buxton et al., 2022; Maruyama et al., 2022), even for plants of high economic, social, and ecological value (Fleming & Muchhala, 2008). Moreover, bats help control insect populations, including pests in agriculture and small vertebrates (Kunz et al., 2011; Ramírez‐Fráncel et al., 2022). Bats are estimated to consume around 3200 caterpillars per hectare per night, amounting to a cost savings of US$ 390.6 million per harvest in Brazil (Aguiar et al., 2021).

Currently, there are 1474 species of bats recognized worldwide (Simmons & Cirranello, 2023), with 184 species occurring in Brazil (Garbino et al., 2022; Lopes et al., 2023; Zortéa et al., 2023), making them the second most diverse order of mammals (Simmons & Cirranello, 2023). This great taxonomic diversity is accompanied by a diversity of feeding habits, including insect consumption, nectar, fruits, seeds, amphibians, fish, small mammals, and even blood (Kunz et al., 2011; Schnitzler & Kalko, 2001). Of these 1470 bat species, 1332 species have been assessed by the International Union for Conservation of Nature (IUCN). They are listed in some of the 11 categories in the Red List of Threatened Species (IUCN Red List). Approximately 17.27% (230) of the species are classified as vulnerable (or extinct—9 species), 6.83% (91) as vulnerable, and 17.72% (236) as data deficient (DD). It is worth noting that one of the criteria for classifying a species as DD is a lack of knowledge about its geographical distribution. According to the IUCN, DD species are those that, although well‐studied and have data on natural history, biology, and ecology, lack information about abundance (Prestonian shortfall) and geographical distribution (Wallacean shortfall).

Both shortfalls should be addressed by increasing efforts to collect primary data. However, fieldwork in remote areas is time‐consuming and costly, with costs varying depending on the region where the work is conducted (Aguiar et al., 2020; Balmford & Gaston, 1999). Furthermore, determining where to allocate efforts for primary data collection remains a key challenge (Aguiar et al., 2020). Nevertheless, the occurrence of a species in a particular area indicates its environmental limits related to abiotic factors and interactions within communities (Pellissier et al., 2010). In this context, species distribution models (SDMs) can address the Wallacean shortfall (Platts et al., 2010; Raxworthy et al., 2003; Razgour et al., 2011). Our goal is to reduce the Wallacean gap among Amazonian bat species. For this, we present (i) a species list of bats sampled in the Pará and Acre states in the last 5 years (2017 to 2022), (ii) the potential distribution of the species considered as new occurrences for the region, (iii) the potential distribution of the DD and NT (IUCN classification) species, and (iv) the comparison of species richness in three sample classes (urban, semi‐urban, and natural).

## 2 MATERIALS AND METHODS

### 2.1 Bats sampling

**FIGURE 1:**
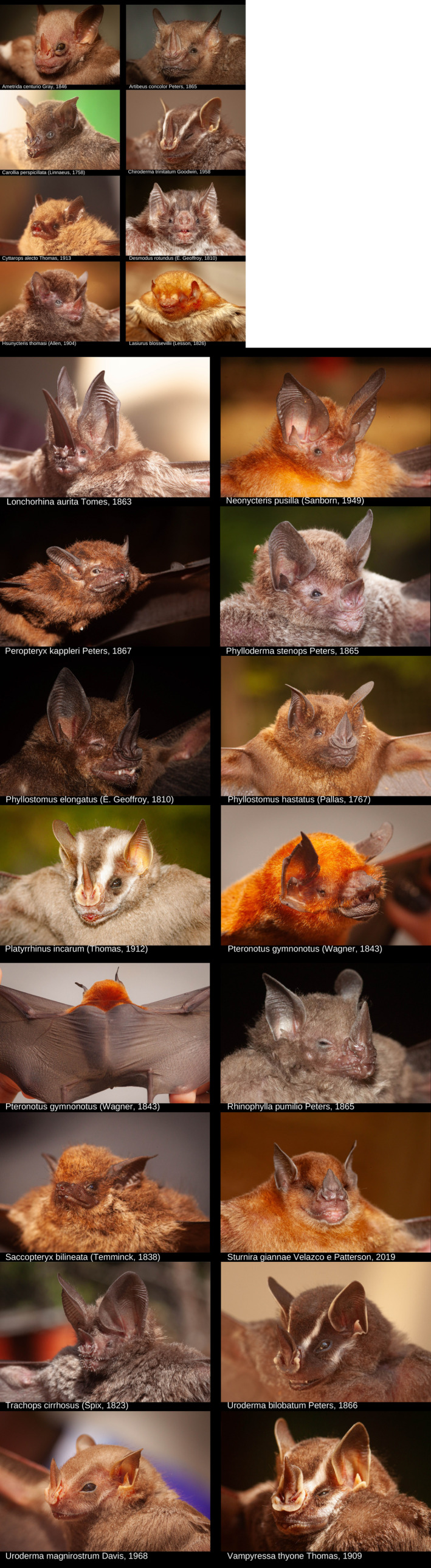
Images of species collected in the states of Pará and Acre. The name of each species is already described in the corresponding image.

**FIGURE 2:**
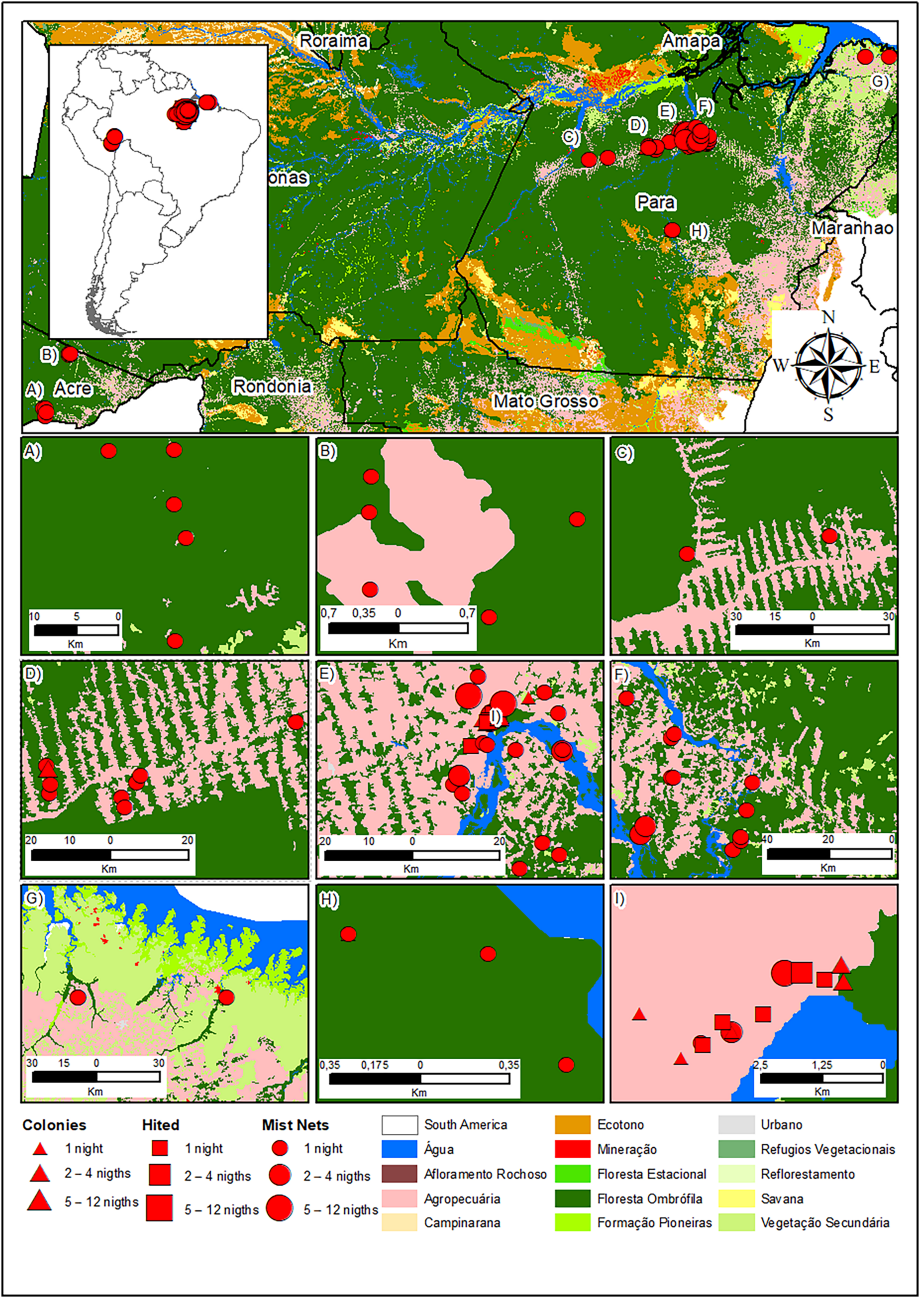
Bat collection points and municipalities.

The bats (Figure 1) were collected in the states of Pará and Acre (Figure 2), reaching 107 nights of mist net in 59 points (Table S1). Additionally, we include nine colonies where bats were registered. The region has a tropical climate of type Am, according to the Köppen climate classification, with an average temperature of 26°C and an average annual rainfall of 1914 mm.

Bats were sampled using mist nets (10 nets measuring 9 x 2.5 m at each point) open at sunset and remaining for 6 h, inspected every half hour. The captured bats were packed in 100% cotton fabric bags. In the field, the individuals were weighed, underwent morphometric measurements, and were also identified by gender, age, and reproductive status. Subsequently, the individuals that were first‐time captures or were not identified to the species level in the field were taken to the Laboratory of Ecology of Altamira (LABECO) of the Federal University of Pará (UFPA), Altamira campus, euthanized by cervical dislocation and the morphometric data (total length of the foot, ear, tragus, forearm, and weight) were measured.

Subsequently, the bats were fixed with 10% formaldehyde and stored in glass containers with 70% alcohol in the ChiroXingu Bat Collection: Nucleus of Studies in Ecology and Conservation of Chiroptera, located at UFPA, Altamira‐PA campus, Brazilian Amazon. The ChiroXingu research group collected the bats from 2017 to 2022 under license 57,294 issued by the Biodiversity Authorization and Information System—SISBIO.

### 2.2 Data analysis

We calculated the sampling effort following the method proposed by Straube and Bianconi (2002). Species richness was estimated by the Jackknife procedure (Heltshe & Forrester, 1983) in the program EstimateS version 8.0 (Colwell, 2005) with 1000 randomizations. Estimated and observed species richness was compared using the 95% confidence intervals. Species richness in the three classes (urban, semi‐urban, and natural) was compared using the intersection among confidence intervals of estimated species richness. We consider only the locations sampled with the mist net to perform the jackknife.

### 2.3 Occurrence data and species modeling

We perform SDM for 22 bat species: *Anoura geoffroyi, Cynomops abrasus, Dermanura anderseni, Diphylla ecaudata, Eumops glaucinus, Eumops perotis, Lasiurus castaneus*, *Lonchophylla mordax, Molossus currentium, Myotis levis, Natalus macrourus Neonycteris pusilla, Peropteryx trinitatis*, *Platyrrhinus angustirostris, Platyrrhinus infuscus, Pygoderma bilabiatum, Saccopteryx gymnure*, *Scleronycteris ega, Thyroptera devivoi*, *Tonatia bidens, Vampyressa pusilla*, and *Vampyressa thyone* (Table S2). We utilized primary data on species collection and occurrence from 1950 onward for the entire Neotropical region, sourced from digital collections such as GBIF (https://www.gbif.org/), SpeciesLink (https://specieslink.net/), and Map of Life (https://mol.org/). Additionally, we conducted searches for articles in digital databases including ISI Web of Science (http://www.webofknowledge.com), Google Scholar (https://scholar.google.com.br/), Scopus (https://www.scopus.com), and the Scientific Electronic Library Online (Scielo, http://www.scielo.org), using each specie name as keywords.

We created potential distribution models, SDMs, for species classified as new occurrences in the region. The distribution data were sourced from Map of Life (https://mol.org/), which compiles biogeographic information and distribution maps for 1784 taxa (Marsh et al., 2022). We also developed SDMs for Data Deficient (DD) and Near Threatened (NT) species, following the classification of the Red List of Threatened Species (IUCN, 2024). These models were created with and without spatial restrictions, following the recommendations of Pimenta et al. (2022), using occurrence points from the entire Neotropical region for bat species included in the study. To minimize overfitting in the models, we filtered the occurrences for each species, avoiding duplicate occurrences and spatial autocorrelation. This procedure involves performing a Moran's correlogram (based on the linear distance between points) and identify and remove occurrences with significant autocorrelation, including the duplicated points. The number of unique occurrences of each species is presented in Table S2.

### 2.4 Environmental variables

We used 19 bioclimatic variables (resolution of 9.4 x 9.4 km) for the neotropical region, obtained from the WorldClim database (http://www.worldclim.org/). These variables include: Mean annual temperature; Monthly mean diurnal temperature range; Isothermality; Temperature seasonality; Maximum temperature of the warmest month; Minimum temperature of the coldest month; Annual temperature range; Mean temperature of the wettest quarter; Mean temperature of the warmest quarter; Mean temperature of the coldest quarter; Annual precipitation; Precipitation of the wettest month; Precipitation of the driest month; Precipitation seasonality; Precipitation of the driest quarter; Precipitation of the wettest quarter; Precipitation of the warmest quarter; Precipitation of the coldest quarter.

The obtained data are part of the group of monthly climate variables sampled between 1970 and 2000 from WorldClim version 2.1 (Fick & Hijmans, 2017). These data are frequently used for species distribution modeling (SDM) to assess the potential distribution of species (Lee & Chen, 2012). To reduce multicollinearity in our dataset, we performed a principal component analysis (PCA) (Legendre & Legendre, 2012) and used the eigenvalues as environmental variables. Then, we selected only the axes that represent an explanation equal to or greater than 95% (De Marco & Nóbrega, 2018), using these axes as model variables.

### 2.5 Algorithms

For the creation of SDMs, four algorithms were used: Maxent (MXE) (Phillips et al., 2017), Random Forest (RDF) (Prasad et al., 2006), Support Vector Machine (SVM) (Guo et al., 2005), and Bayesian Gaussian (GAU) (Golding and Purse, 2016). An ensemble combining the final suitability maps was generated by the four algorithms to minimize model uncertainties (Araújo and New, 2007; Diniz‐Filho et al., 2009). To mitigate model uncertainties, an ensemble approach was adopted as the final model (Pimenta et al., 2022; Velazco et al., 2019). This ensemble model consists of the average suitability across models for which the Jaccard threshold values (Pimenta et al., 2022) were greater than the average thresholds for each species (Velazco et al., 2019). The Jaccard threshold was selected to minimize omission and commission errors in the models (Pimenta et al., 2022). Additionally, we applied spatial restrictions to the models to minimize overprediction in distribution models (Mendes et al., 2020; Pimenta et al., 2022). To do this, we created a binary occurrence map, where suitability values greater than the Jaccard threshold indicated species presence, and then partitioned it into pixels with species occurrence and pixels without species occurrence.

Subsequently, only pixels where the species was predicted and had species records or pixels where the species was predicted and were near pixels with predictions and occurrence points were retained in the species' potential distribution map (Pimenta et al., 2022). For the partitioning of the binary map, we considered two methods: (i) Species with more than 30 occurrence points: partitioning the map using the chessboard method (Andrade et al., 2020); (ii) Species with fewer than 30 points: randomly selecting a percentage of points for modeling and another for evaluation, with 70% of the points selected for the model and 30% for evaluation (Pimenta et al., 2022). Since spatial restriction generates more conservative maps, limiting the occurrence areas to locations near or with species occurrence, we conducted a second modeling without spatial restriction. Thus, we have a more restrictive and conservative model (a model with spatial restriction) and a less conservative model containing areas with environmental suitability without considering whether the species occurs or not. All procedures were performed using the enmtml function implemented in the ENMTL package (Andrade et al., 2020) for the R environment (R Core Team, 2010).

### 2.6 Model evaluation

The evaluation was performed using Receiver Operating Characteristic (ROC) curves, and the efficiency of each model was assessed using the True Skill Statistic (TSS) test, which has been widely advocated as an appropriate discrimination metric that is independent of prevalence (Allouche et al., 2006; Shabani et al., 2018). TSS is an intuitive method for measuring the performance of Species Distribution Models (SDMs), which calculates sensitivity (true positive rate, TPR) and specificity (true negative rate, TNR) values, where predictions are expressed as presence‐absence maps. This test slightly restricts the occurrence area, leading to a less inclusive map, considering errors of omission in species distribution (false negatives) and commission (false positives), with values ranging from −1 to +1 (Sensitivity + Specificity) to indicate the predictive ability of the models. Models with TSS values close to +1 reflect good predictive ability; models with TSS values between 0.2 and 0.6 are considered fair to moderate; and models with TSS values close to 0 or negative indicate low capability.

However, TSS values can be misleading when the number of true negatives assigns higher values to species with lower prevalence (Lawson et al., 2014). To avoid these deficiencies, we propose focusing the evaluation metrics on three components of the confusion matrix: true positives, false positives, and false negatives, neglecting true negatives that could inflate the data. In other words, we aim to maximize true positives while minimizing false positives and false negatives relative to true positives (Leroy et al., 2018).

## 3 RESULTS

**FIGURE 3:**
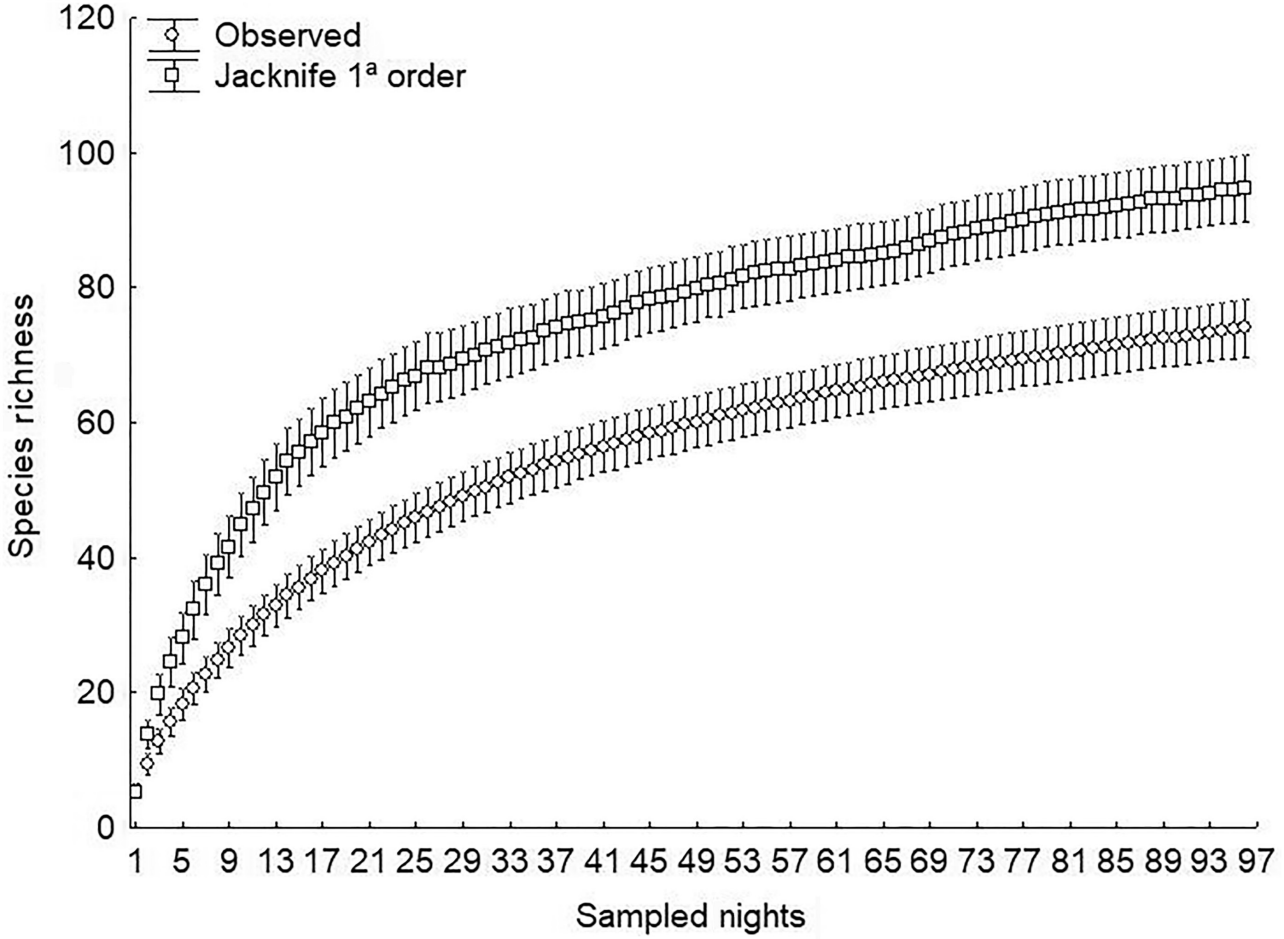
Observed and estimated bat species richness sampled between 2017 and 2022. The bars represent the 95% confidence interval.

**FIGURE 4:**
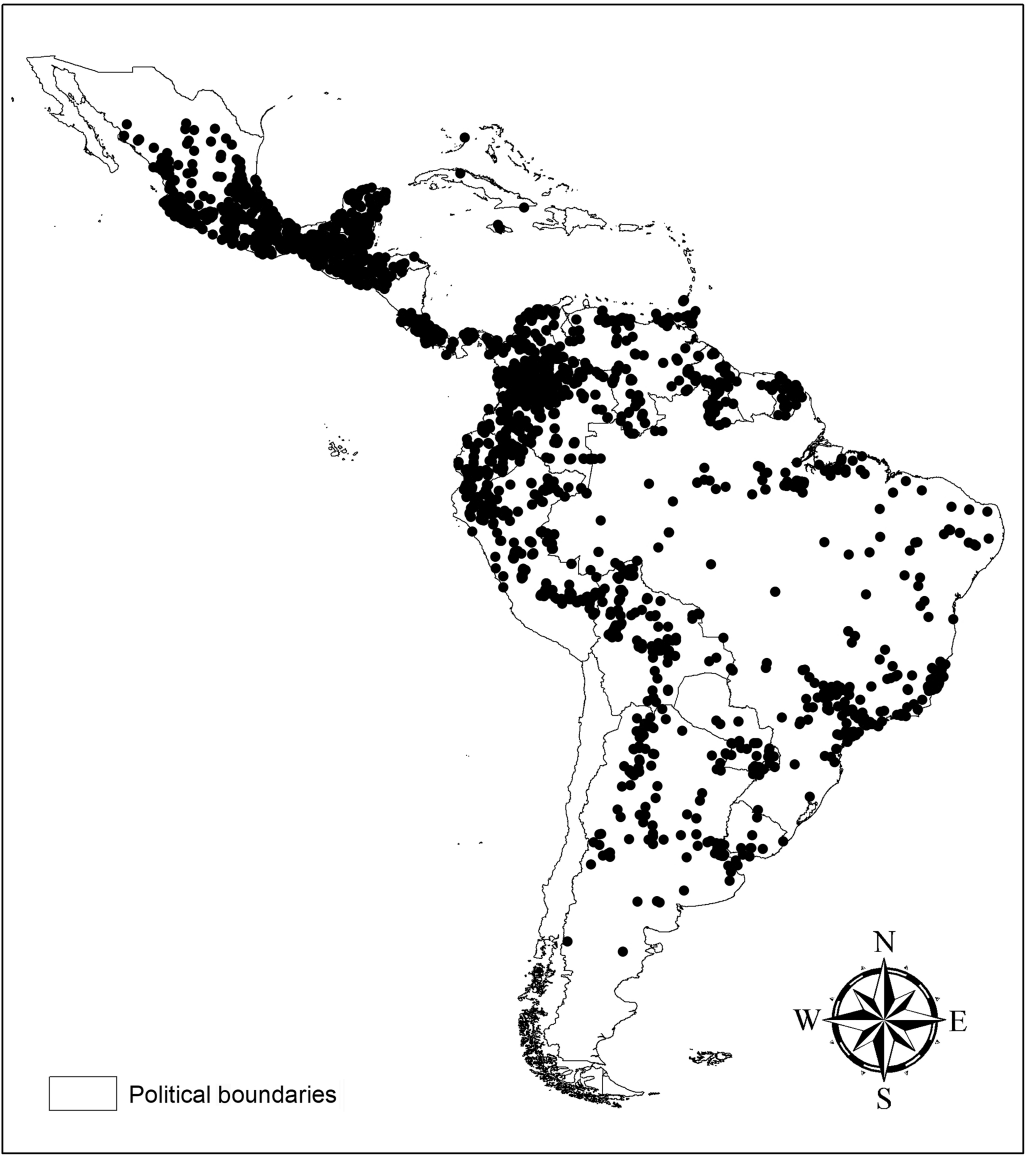
Occurrences included in the species distribution modeling. The procedures of modeling are described in the Appendix S1 and the evaluation of models are in the Table S3.

With 96 nights of collection and 129,600 square meters of mist netting, we obtained 75 bat species (Table S2), with an estimated 94.78 species (Figure 3). Six species (*Eumops glaucinus, Eumops perotis, Molossus coibensis, Molossus currentium, Nyctinomops laticaudatus*, and *Natalus macrourus*) were manually sampled in colonies. One species (*Thyroptera discifera*) was recorded only from roadkill, totaling 82 species and representing all nine bat families found in Brazil (Emballonuridae, Furipteridae, Molossidae, Mormoopidae, Natalidae, Noctilionidae, Phyllostomidae, Thyropteridae, and Vespertilionidae) (Table S2), with the family Phyllostomidae being the most diverse and abundant. Eight species are classified as Data Deficient (DD) (*Peropteryx trinitatis*, *Cynomops abrasus, Scleronycteris ega, Neonycteris pusilla, Tonatia bidens, Vampyressa pusilla, Thyroptera devivoi, and Lasiurus castaneus*), two as Near Threatened (NT) (*Natalus macrourus and Lonchophylla mordax*), and 21 species are considered range extensions (*Anoura geoffroyi, Dermanura anderseni, Diphylla ecaudata, Eumops glaucinus, Eumops perotis, Lasiurus castaneus, Lonchophylla mordax, Molossus currentium, Myotis levis, Natalus macrourus, Neonycteris pusilla, Platyrrhinus angustirostris, Platyrrhinus infuscus, Pygoderma bilabiatum, Saccopteryx gymnure*, *Scleronycteris ega*, *Thyroptera devivoi*, *Tonatia bidens*, *Trinycteris nicefori*, *Vampyressa pusilla*, and *Vampyressa thyone*) (Table S2). For the SDMs of DD, NT, and range extension species, we used 2612 occurrence points distributed throughout the Neotropical region (Table S2, Figure 4), with species ranging from five unique occurrence points (*Lasiurus castaneus* and *Neonycteris pusilla*) to 786 points (*Anoura geoffroyi*). The SDMs showed significant variation in terms of distribution areas, with the areas of SDMs with geographic restrictions tending to be smaller than those without restrictions. Some models exhibited limited potential distribution areas for the sampled Amazon region (Figures [Link], [Link], [Link], [Link], [Link], [Link]), with three SDMs showing restricted or non‐occurrence areas for the sampled Amazon region (Figures S8, S15, S17, and S18).

## 4 DISCUSSION

The high diversity of Phyllostomidae bats, a relatively common pattern in studies conducted with mist nets in the Neotropical region, can be explained by the very use of the capture method, a methodology known to be selective. This may explain the abundance of the family in our database (Pedro & Taddei, 1997; Sipinski & Reis, 1995).

We observed different environments, ranging from urban and rural areas to natural habitats, but despite the high diversity of bats in our study, it would still be possible to add new species to the list, especially if we use other sampling methodologies, such as acoustic monitoring. This way we could capture other families that are not normally captured in mist nets.

Supporting this, we captured six species, five from the Molossidae family (*Eumops glaucinus*, *Eumops perotis*, *Molossus coibensis*, *Molossus currentium*, and *Nyctinomops laticaudatus*) and one from the Natalidae (*Natalus macrourus*) that were sampled only through active capture when we located their shelters and manually captured them. Additionally, one species, *Thyroptera discifera*, was recorded from a single individual donated to the laboratory and was not captured with mist nets or active search.

The species richness estimator predicts these additions to the species list (we recorded 75 and estimated the occurrence of nearly 95 species) based solely on mist net data. Additionally, 28% (21 species) of the captured species are considered range extensions, supporting that work with primary biogeographic data are still necessary today, especially in the Brazilian Amazon region.

The Brazilian Amazon region has a vast geographic expanse and few consolidated research centers (Aguiar et al., 2020; Delgado‐Jaramillo et al., 2020). This pattern is observed not only for bats but also for other biological groups (Delgado‐Jaramillo et al., 2020; Dias‐Silva et al., 2021). We observe major research centers concentrated in the southwestern and coastal regions of Brazil, with few research groups in the interior of Brazil, including the Amazon Biome and the Caatinga (Brito et al., 2009; Delgado‐Jaramillo et al., 2020; Lewinsohn & Prado, 2002).

Studies investigating priority areas for the conservation of biological groups tend to rank the Caatinga region as less critical for conservation (Dias‐Silva et al., 2021). However, this result is not due to its actual low importance but rather because of the failure of species distribution models (SDMs) to predict occurrences in these areas. This failure results from a need for primary biogeographic data (Delgado‐Jaramillo et al., 2020; Silva et al., 2018). This absence can be observed in the distribution maps of data points used for these SDMs. This gap is particularly evident in the SDM of Myotis levis, where the species has three occurrence points for Pará (our study area), with one reported by us and many points concentrated in the South American region. This concentration tends to generate an SDM that does not include the study area, even though there are (few) species records in the region.

Some other less obvious examples of the lack of primary biogeographic data are observed for the species *Natalus macrourus*, *Diphylla ecaudata*, *Anoura geoffroyi*, *Pygoderma bilabiatum*, *Platyrrhinus infuscus*, and *Dermanura anderseni*. Despite the SDMs showing suitable areas for the species in the region, these areas are limited to small “islands” near the collected species. These gaps in the geographic distribution of species are, in many cases, the result of a lack of primary biogeographic data. This highlights the need for investments in collecting primary data for emerging groups in regions with little sampling. The lack of primary biogeographical data and uncertainties regarding the geographical distribution of species have been observed in other bat species, such as *Tonatia maresi* (Aguiar et al., 2015), *Histiotus velatus* (Da Silva et al., 2021), and more recently in *Tadarida brasiliensis* brasiliensis (do Amaral et al., 2023). Furthermore, changes in the species' distribution areas over the years due to the addition of new occurrence points to models have also been noted (Da Silva et al., 2021), thus reinforcing the need for investments in obtaining primary biogeographical data (Aguiar et al., 2015; Da Silva et al., 2021).

For the Amazon region, in particular, collecting biogeographic data is very costly because transportation is mainly done by river or on poorly maintained roads that become impassable during specific periods of the year. In the region analyzed in this study, along the Trans‐Amazonian Highway, there is a period of 4–5 months (from December to April/May) when the roads are practically impassable, including the main highway, BR 230. This means sampling is concentrated during the dry season or in areas closer to the research center where we operate (Aguiar et al., 2020; Dias‐Silva et al., 2021).

We want to emphasize two points regarding the reduction of the Wallacean gap. The first point pertains to bat species with expanded geographic distributions, with 21 out of the 75 sampled species falling into this category. This result highlights that the areas where we focused our studies, especially those in Pará, are areas with poorly known bat fauna. The study area we explored is highly diverse and potentially harbors species that are new to the literature (Linnaean gap), as we have observed the recent description (after 2010) of six bat species (Aguiar et al., 2020). Furthermore, the number of sampling points in the studied region is low compared to the central portion of the Atlantic Forest (Aguiar et al., 2020), underscoring the need for inventories and studies that generate primary data.

The other point to be noted, related to the Wallacean shortfall, concerns the environmental similarity between the sampled points in our study and the areas with the highest sampling efforts in Brazil. Areas with low bat sampling, especially those in the Amazon biome, have low environmental similarity to Brazil's more heavily sampled areas (Aguiar et al., 2020). Therefore, areas considered as sampling gaps may be areas of occurrence for new species, both in terms of species already described but with geographic distributions that do not include Brazil and species new to science.

This argument can be supported by the expanded distribution of *Platyrrhinus guianensis* Velazco and Lim, 2014 (Lopes et al., 2023), *Artibeus amplus* Handley, 1987 (Zortéa et al., 2023), and *Choeroniscus godmani* (Thomas, 1903) (Garbino et al., 2022). All these species are considered new occurrences for Brazil, with *P. guianensis* and *A. amplus* having a single occurrence point for Brazil, located in the Amazon region near our study area in Pará (Lopes et al., 2023; Zortéa et al., 2023). Additionally, the species *P. guianensis* was described in 2014, reinforcing the idea that the region sampled in our study is potentially a diverse area and a potential source of new species unknown to science (Linnaean shortfall).

The environmental difference between the areas considered as sampling gaps for bats in Brazil and well‐sampled locations (Aguiar et al., 2020), measured based on the bioclimatic conditions used in SDM (Bendjeddou et al., 2022; Da Silva et al., 2021; Da Silva et al., 2022; Delgado‐Jaramillo et al., 2020; Pimenta et al., 2022), could explain why the distribution models presented here do not predict species occurrence in the region, even though the species is captured on‐site. Indeed, the failure of our models to predict occurrence may be a combination of limited sampling in the region, along with occurrence points that are concentrated in well‐sampled areas with different environmental conditions than our study area.

Occurrence points centered in environmentally similar areas, contrasting with a few points with low environmental similarity, can lead to models with over‐prediction of the distribution area, mainly when geographic restriction is applied (Pimenta et al., 2022; Soberón & Nakamura, 2009). Consequently, models may suggest that areas with few occurrence points (and low environmental similarity compared to the more extensive set of points, in the case of Brazilian bats) have a low probability of species occurrence and are considered unsuitable for the species (Chang et al., 2020; Peterson et al., 2008; Soberón & Nakamura, 2009).

However, the need for more suitability might be due to the environmental difference between the areas where occurrence points are concentrated and where the species has an extended range. This scenario is observed in some of the presented occurrence extension models, where many points are located in environmentally dissimilar areas from the new occurrence point (Aguiar et al., 2020). Consequently, besides having a large sampling gap for bats, the Amazon region might have its diversity overestimated by species distribution models (SDMs). This sampling gap has a direct impact on species conservation prioritization efforts since some methods are derived from SDMs (e.g., Fielding & Bell, 1997; Meggs et al., 2004; Silva et al., 2018), potentially leading to the creation of inefficient reserves for biodiversity conservation (Brasil et al., 2021; Dias‐Silva et al., 2021).

We hope the SDMs can serve as a foundation or be directly used to guide conservation efforts, especially for DD and NT species. Additionally, 21 species classified as new occurrences should be seen as a reminder that primary biogeographic data, including mist‐netting and other sampling methods, are still necessary for the Neotropical region. Investments in such research in the Amazon and Caatinga biomes are crucial, mainly when directed toward small emerging research groups located outside the state capitals.

## AUTHOR CONTRIBUTIONS

**Thiago Bernardi Vieira:** Conceptualization (lead); data curation (lead); formal analysis (lead); funding acquisition (lead); investigation (lead); methodology (lead); project administration (lead); software (lead); supervision (lead); validation (lead); visualization (lead); writing – original draft (lead); writing – review and editing (lead). **Rafaela Jemely Rodrigues Alexandre:** Data curation (equal); methodology (equal); writing – review and editing (equal). **Simone Almeida Pena:** Data curation (equal); methodology (equal); writing – review and editing (equal). **Letícia Lima Correia:** Data curation (equal); investigation (equal); methodology (equal); writing – review and editing (equal). **Ariane de Sousa Brasil:** Investigation (equal); methodology (equal); writing – review and editing (equal). **Ludmilla Moura de Souza Aguiar:** Formal analysis (equal); methodology (equal); validation (equal); writing – review and editing (equal). **Paulo De Marco:** Formal analysis (equal); software (equal); writing – review and editing (equal). **Albert David Ditchfield:** Formal analysis (equal); methodology (equal); validation (equal); writing – review and editing (equal).

## FUNDING INFORMATION

This research benefited from resources from Vale SA's environmental compensation administered by the Centro Nacional de Pesquisa e Conservação de Cavernas (Cecav/ICMBio) and services to the Brazilian Society for the Study of Chiropterans, SBEQ, as part of the DD Program, The Species More Unknown in Brazil, and with resources from the Termo de Compromisso de Compensação Espeleológica, TCCE VALE 1/2018, Edital Ferruginosas 01/2021, under the administration of the Instituto Brasileiro de Desenvolvimento e Sustentabilidade, IABS. National Council for Scientific and Technological Development, CNPQ. Amazon Foundation to Support Studies and Research—FAPESPA. Foundation Coordination for the Improvement of Higher Education Personnel—CAPES.

## CONFLICT OF INTEREST STATEMENT

There is no conflict of interest.

## Supporting information


Table S1.‐S3.

Appendix S1.

Figure S1.

Figure S2.

Figure S3.

Figure S4.

Figure S5.

Figure S6.

Figure S7.

Figure S8.

Figure S9.

Figure S10.

Figure S11.

Figure S12.

Figure S13.

Figure S14.

Figure S15.

Figure S16.

Figure S17.

Figure S18.

Figure S19.

Figure S20.

Figure S21.

Figure S22.

Figure S23.

Figure S24.

## Data Availability

All information about the data used in this work, how it was done and in what form is distributed throughout the text or in the supplementary material.

## References

[ece311392-bib-0001] Aguiar, L. M. S. , Bueno‐Rocha, I. D. , Oliveira, G. , Pires, E. S. , Vasconcelos, S. , Nunes, G. L. , Frizzas, M. R. , & Togni, P. H. B. (2021). Going out for dinner—The consumption of agriculture pests by bats in urban areas. PLoS One, 16(10), e0258066. 34673777 10.1371/journal.pone.0258066 PMC8530310

[ece311392-bib-0002] Aguiar, L. M. S. , da Rosa, R. O. L. , Jones, G. , & Machado, R. B. (2015). Effect of chronological addition of records to species distribution maps: The case of Tonatia saurophila maresi (Chiroptera, Phyllostomidae) in South America. Austral Ecology, 40(7), 836–844.

[ece311392-bib-0003] Aguiar, L. M. S. , Pereira, M. J. R. , Zortéa, M. , & Machado, R. B. (2020). Where are the bats? An environmental complementarity analysis in a megadiverse country. Diversity and Distributions, 26(11), 1510–1522.

[ece311392-bib-0101] Allouche, O. , Tsoar, A. , & Kadmon, R. (2006). Assessing the accuracy of species distribution models: Prevalence, kappa and the true skill statistic (TSS). Journal of Applied Ecology, 43, 1223–1232. 10.1111/j.1365-2664.2006.01214.x

[ece311392-bib-0102] Andrade, A. F. A. , Velazco, S. J. E. , & De Marco Júnior, P. (2020). ENMTML: An R package for a straightforward construction of complex ecological niche models. Environmental Modelling and Software, 125, 104615. 10.1016/j.envsoft.2019.104615

[ece311392-bib-0103] Araújo, M. B. , & New, M. (2007). Ensemble forecasting of species distributions. Trends in Ecology & Evolution, 22, 42–47. 10.1016/j.tree.2006.09.010 17011070

[ece311392-bib-0004] Balmford, A. , & Gaston, K. J. (1999). Why biodiversity surveys are good value. Nature, 398(6724), 204–205.

[ece311392-bib-0005] Baqi, A. , Lim, V. , Yazid, H. , Anwarali Khan, F. A. , Lian, C. J. , Nelson, B. R. , Sathiya Seelan, J. S. , Appalasamy, S. , Mokhtar, S. I. , & Kumaran, J. V. (2022). A review of durian plant‐bat pollinator interactions. Journal of Plant Interactions, 17(1), 105–126.

[ece311392-bib-0006] Bendjeddou, M. L. , Bouam, I. , Aulagnier, S. , Abdelaziz, S. E. , Etayeb, K. , Mihalca, A. D. , & Sándor, A. D. (2022). First record of the lesser horseshoe bat, *Rhinolophus hipposideros* (Borkhausen, 1797), in Libya and potential distribution in North Africa. Mammalia, 86(4), 328–332.

[ece311392-bib-0007] Bernard, E. , Aguiar, L. M. , Brito, D. , Cruz‐Neto, A. P. , Gregorin, R. , Machado, R. B. , Oprea, M. , Paglia, A. P. , & Tavares, V. C. (2012). Uma análise de horizontes sobre a conservação de morcegos no Brasil. Mamíferos Do Brasil: Genética, Sistemática, Ecologia e Conservação, 2, 19–35.

[ece311392-bib-0008] Brasil, L. S. , Andrade, A. F. A. , Ribeiro, B. R. , Spigoloni, Z. A. , Juen, L. , & De Marco, P. (2021). A niche‐based gap analysis for the conservation of odonate species in the Brazilian Amazon. Aquatic Conservation: Marine and Freshwater Ecosystems, 31(5), 1150–1157.

[ece311392-bib-0009] Brasileiro, L. A. M. , Machado, R. B. , & Aguiar, L. M. S. (2022). Ecosystems services provided by bats are at risk in Brazil. Frontiers in Ecology and Evolution, 10, 852177.

[ece311392-bib-0010] Brito, D. , Oliveira, L. C. , Oprea, M. , & Mello, M. A. (2009). An overview of Brazilian mammalogy: Trends, biases and future directions. Zoologia (Curitiba), 26, 67–73.

[ece311392-bib-0011] Buxton, M. N. , Gaskett, A. C. , Lord, J. M. , & Pattemore, D. E. (2022). A global review demonstrating the importance of nocturnal pollinators for crop plants. Journal of Applied Ecology, 59(12), 2890–2901.

[ece311392-bib-0012] Chang, Y. L. , Xia, Y. , Peng, M. W. , Chu, G. M. , & Wang, M. (2020). Maxent modelling for predicting impacts of climate change on the potential distribution of anabasis aphylla in northwestern China. Applied Ecology and Environmental Research, 18(1), 1637–1648.

[ece311392-bib-0013] Colwell, R. K. (2005). EstimateS: Statistical estimation of species richness and shared species from samples . Version 7.5. http://purl.oclc.org/estimates

[ece311392-bib-0014] Da Silva, L. C. , Almeida, R. G. , da Silva, P. H. , Oprea, M. , Mendes, P. , Brito, D. , & Bernardi Vieira, T. (2021). Temporal changes in the potential geographic distribution ofHistiotus velatus (Chiroptera, Vespertilionidae), the “decade effect”. Ecology and Evolution, 11(23), 16972–16980. 34938485 10.1002/ece3.8333 PMC8668744

[ece311392-bib-0015] Da Silva, L. C. N. , Vieira, T. B. , Oliveira, A. S. Q. A. , Mendes, P. , Peixoto, F. P. , Oprea, M. , & de Aguiar, L. M. S. (2022). Bat species of a karstic region in the Brazilian savanna and extension of the *Hsunycteris thomasi* (Phyllostomidae: Lonchophyllinae) distribution. Mammalia, 86(1), 27–36.

[ece311392-bib-0016] De Marco, P. , & Nóbrega, C. C. (2018). Evaluating collinearity effects on species distribution models: An approach based on virtual species simulation. PLoS One, 13, e0202403. 30204749 10.1371/journal.pone.0202403 PMC6133275

[ece311392-bib-0017] Delgado‐Jaramillo, M. , Aguiar, L. M. S. , Machado, R. B. , & Bernard, E. (2020). Assessing the distribution of a species‐rich group in a continental‐sized megadiverse country: Bats in Brazil. Diversity and Distributions, 26(5), 632–643.

[ece311392-bib-0018] Dias‐Silva, K. , Vieira, T. B. , Moreira, F. F. F. , Juen, L. , & Hamada, N. (2021). Protected areas are not effective for the conservation of freshwater insects in Brazil. Scientific Reports, 11(1), 21247. 34711886 10.1038/s41598-021-00700-0 PMC8553851

[ece311392-bib-0019] Diniz‐Filho, J. A. F. , Loyola, R. D. , Raia, P. , Mooers, A. O. , & Bini, L. M. (2013). Darwinian shortfalls in biodiversity conservation. Trends in Ecology & Evolution, 28(12), 689–695. 24091208 10.1016/j.tree.2013.09.003

[ece311392-bib-0104] Diniz‐Filho, J. A. F. , Mauricio Bini, L. , Fernando Rangel, T. , Loyola, R. D. , Hof, C. , Nogués‐Bravo, D. , & Araújo, M. B. (2009). Partitioning and mapping uncertainties in ensembles of forecasts of species turnover under climate change. Ecography (Cop.), 32, 897–906. 10.1111/j.1600-0587.2009.06196.x

[ece311392-bib-0020] do Amaral, I. S. , Bandeira Pereira, J. , Vancine, M. H. , Morales, A. E. , Althoff, S. L. , Gregorin, R. , Pereira, M. J. R. , Valiati, V. H. , & Rosa de Oliveira, L. (2023). Where do they live? Predictive geographic distribution of *Tadarida brasiliensis* brasiliensis (Chiroptera, Molossidae) in South America. Neotropical Biology & Conservation, 18(3), 139–156.

[ece311392-bib-0021] dos Santos, A. J. , Vieira, T. B. , & Faria, K. C. (2016). Effects of vegetation structure on the diversity of bats in remnants of Brazilian Cerrado savanna. Basic and Applied Ecology, 17(8), 720–730.

[ece311392-bib-0022] Estrada, A. , & Coates‐Estrada, R. (2002). Bats in continuous forest, forest fragments and in an agricultural mosaic habitat‐Island at Los Tuxtlas, Mexico. Biological Conservation, 103(2), 237–245.

[ece311392-bib-0023] Fick, S. E. , & Hijmans, R. J. (2017). WorldClim 2: New 1‐km spatial resolution climate surfaces for global land areas. International Journal of Climatology, 37, 4302–4315.

[ece311392-bib-0024] Fielding, A. H. , & Bell, J. F. (1997). A review of methods for the assessment of prediction errors in conservation presence/absence models. Environmental Conservation, 24(1), 38–49.

[ece311392-bib-0025] Figueiró, A. (2015). Biogeografia: Dinâmicas e transformações da natureza. Oficina de Textos.

[ece311392-bib-0026] Fleming, T. H. , & Muchhala, N. (2008). Nectar‐feeding bird and bat niches in two worlds: Pantropical comparisons of vertebrate pollination systems. Journal of Biogeography, 35(5), 764–780.

[ece311392-bib-0027] Garbino, G. S. T. , Brandão, M. V. , & da Cunha Tavares, V. (2022). First confirmed records of Godman's long‐tailed bat, *Choeroniscus godmani* (Thomas, 1903) (Chiroptera, Phyllostomidae), from Brazil and Panama. Check List, 18(3), 493–499.

[ece311392-bib-0028] Gatti, L. V. , Basso, L. S. , Miller, J. B. , Gloor, M. , Gatti Domingues, L. , Cassol, H. L. G. , Tejada, G. , Aragão, L. E. O. C. , Nobre, C. , Peters, W. , Marani, L. , Arai, E. , Sanches, A. H. , Corrêa, S. M. , Anderson, L. , Von Randow, C. , Correia, C. S. C. , Crispim, S. P. , & Neves, R. A. L. (2021). Amazonia as a carbon source linked to deforestation and climate change. Nature, 595(7867), 388–393. 34262208 10.1038/s41586-021-03629-6

[ece311392-bib-0105] Golding, N. , & Purse, B. V. (2016). Fast and flexible Bayesian species distribution modelling using Gaussian processes. Methods in Ecology and Evolution, 7, 598–608. 10.1111/2041-210X.12523

[ece311392-bib-0029] Gomez, V. , Beuchle, R. , Shimabukuro, Y. , Grecchi, R. , Simonetti, D. , Eva, H. D. , & Achard, F. (2015). A long‐term perspective on deforestation rates in the Brazilian Amazon. The International Archives of the Photogrammetry, Remote Sensing and Spatial Information Sciences, 40, 539–544.

[ece311392-bib-0030] Guo, Q. , Kelly, M. , & Graham, C. H. (2005). Support vector machines for predicting distribution of sudden oak death in California. Ecological Modelling, 182, 75–90.

[ece311392-bib-0031] Heltshe, J. F. , & Forrester, N. E. (1983). Estimating species richness using the jackknife procedure. Biometrics, 39, 1–11. 6871338

[ece311392-bib-0032] Hutson, A. M. , Mickleburgh, S. P. , & Racey, P. A. (2001). Global status survey and conservation action plan. Microchiropteran bats (p. 254). Information Press.

[ece311392-bib-0033] Kasso, M. , & Balakrishnan, M. (2013). Ecological and economic importance of bats (order Chiroptera). ISRN Biodiversity, 2013, 1–9.

[ece311392-bib-0106] IUCN . (2024). The IUCN Red List of Threatened Species. Version 2023‐1. https://www.iucnredlist.org

[ece311392-bib-0034] Kunz, T. H. , Braun de Torrez, E. , Bauer, D. , Lobova, T. , & Fleming, T. H. (2011). Ecosystem services provided by bats. Annals of the new York Academy of Sciences, 1223(1), 1–38. 21449963 10.1111/j.1749-6632.2011.06004.x

[ece311392-bib-0035] Kunz, T. H. , & Fenton, M. B. (2005). Bat ecology , Melville B. ed. Chicago and London.

[ece311392-bib-0107] Lawson, C. R. , Hodgson, J. A. , Wilson, R. J. , & Richards, S. A. (2014). Prevalence, thresholds and the performance of presence‐absence models. Methods in Ecology and Evolution, 5, 54–64. 10.1111/2041-210X.12123

[ece311392-bib-0036] Lee, M. R. , & Chen, T. T. (2012). Revealing research themes and trends in knowledge management: From 1995 to 2010. Knowledge‐Based Systems, 28, 47–58.

[ece311392-bib-0037] Legendre, P. , & Legendre, L. (2012). Numerical ecology. Elsevier.

[ece311392-bib-0108] Leroy, B. , Delsol, R. , Hugueny, B. , Meynard, C. N. , Barhoumi, C. , Barbet‐Massin, M. , & Bellard, C. (2018). Without quality presence–absence data, discrimination metrics such as TSS can be misleading measures of model performance. Journal of Biogeography, 45, 1994–2002. 10.1111/jbi.13402

[ece311392-bib-0038] Lewinsohn, T. , & Prado, P. I. (2002). Biodiversidade brasileira: Síntese do estado atual do conhecimento (pp. 17–25). Editora Contexto.

[ece311392-bib-0039] Lobo, J. M. , Hortal, J. , Yela, J. L. , Millán, A. , Sánchez‐Fernández, D. , García‐Roselló, E. , González‐Dacosta, J. , Heine, J. , González‐Vilas, L. , & Guisande, C. (2018). KnowBR: An application to map the geographical variation of survey effort and identify well‐surveyed areas from biodiversity databases. Ecological Indicators, 91, 241–248.

[ece311392-bib-0040] Lomolino, M. V. (2004). Conservation biogeography. In M. V. Lomolino & L. R. Heaney (Eds.), Frontiers of Biogeography: New Directions in the Geography of Nature (p. 293). Sinauer.

[ece311392-bib-0041] Lopes, G. P. , Oliveira, R. C. , Santos, T. C. M. , Velazco, P. M. , Bobrowiec, P. E. D. , Silva, M. N. F. , Hrbek, T. , & Farias, I. P. (2023). First record of *Platyrrhinus guianensis* Velazco and Lim, 2014 (Chiroptera, Phyllostomidae) for Brazil. Mammalia, 7068, 591–594.

[ece311392-bib-0042] López‐Baucells, A. , Torrent, L. , Rocha, R. , Bobrowiec, P. E. , Palmeirim, J. M. , & Meyer, C. F. (2019). Stronger together: Combining automated classifiers with manual post‐validation optimizes the workload vs reliability trade‐off of species identification in bat acoustic surveys. Ecological Informatics, 49, 45–53.

[ece311392-bib-0044] Marsh, C. J. , Sica, Y. V. , Burgin, C. J. , Dorman, W. A. , Anderson, R. C. , del Toro Mijares, I. , Vigneron, J. G. , Barve, V. , Dombrowik, V. L. , Duong, M. , Guralnick, R. , Hart, J. A. , Maypole, J. K. , McCall, K. , Ranipeta, A. , Schuerkmann, A. , Torselli, M. A. , Lacher, T. , Mittermeier, R. A. , … Jetz, W. (2022). Expert range maps of global mammal distributions harmonised to three taxonomic authorities. Journal of Biogeography, 49(5), 979–992. 35506011 10.1111/jbi.14330 PMC9060555

[ece311392-bib-0045] Maruyama, P. K. , Silva, J. L. S. , Gomes, I. N. , Bosenbecker, C. , Cruz‐Neto, O. , Oliveira, W. , Cardoso, J. C. F. , Stewart, A. B. , & Lopes, A. V. (2022). A global review of urban pollinators and implications for maintaining pollination services in tropical cities. In F. Angeoletto , P. Tryjanowski , & M. Fellowes (Eds.), Ecology of tropical cities: Natural and social sciences applied to the conservation of urban biodiversity (pp. 1–29). Springer Nature.

[ece311392-bib-0046] Meggs, J. M. , Munks, S. A. , Corkrey, R. , & Richards, K. (2004). Development and evaluation of predictive habitat models to assist the conservation planning of a threatened lucanid beetle, *Hoplogonus simsoni*, in north‐east Tasmania. Biological Conservation, 118(4), 501–511.

[ece311392-bib-0111] Mendes, P. , Velazco, S. J. E. , de Andrade, A. F. A. , & De Marco, P. (2020). Dealing with overprediction in species distribution models: How adding distance constraints can improve model accuracy. Ecological Modelling, 431, 109180. 10.1016/j.ecolmodel.2020.109180

[ece311392-bib-0047] Muscarella, R. , & Fleming, T. H. (2007). The role of frugivorous bats in tropical forest succession. Biological Reviews, 82(4), 573–590. 17944618 10.1111/j.1469-185X.2007.00026.x

[ece311392-bib-0048] Nóbrega, C. C. , & De Marco Jr, P. (2011). Unprotecting the rare species: A niche‐based gap analysis for odonates in a core Cerrado area. Diversity and Distributions, 17(3), 491–505.

[ece311392-bib-0049] Palheta, L. R. , Urbieta, G. L. , Brasil, L. S. , Dias‐Silva, K. , da Silva, J. B. , Graciolli, G. , Aguiar, L. M. S. , & Vieira, T. B. (2020). The effect of urbanization on bats and communities of bat flies (Diptera: Nycteribiidae and Streblidae) in the Amazon, northern Brazil. Acta Chiropterologica, 22(2), 403–416.

[ece311392-bib-0050] Pedro, W. A. , & Taddei, V. A. (1997). Taxonomic assemblage of bats from Panga reserve, southeastern Brazil: Abundance patterns andtrophic relations in the Phyllostomidae (Chiroptera). Boletin Do Museu de BiologiaMello Leitão, 6, 3–21.

[ece311392-bib-0051] Pellissier, L. , Anne Bråthen, K. , Pottier, J. , Randin, C. F. , Vittoz, P. , Dubuis, A. , Yoccoz, N. G. , Alm, T. , Zimmermann, N. E. , & Guisan, A. (2010). Species distribution models reveal apparent competitive and facilitative effects of a dominant species on the distribution of tundra plants. Ecography, 33(6), 1004–1014.

[ece311392-bib-0052] Peterson, A. T. , Papeş, M. , & Soberón, J. (2008). Rethinking receiver operating characteristic analysis applications in ecological niche modeling. Ecological Modelling, 213(1), 63–72.

[ece311392-bib-0053] Phillips, S. J. , Anderson, R. P. , Dudík, M. , Schapire, R. E. , & Blair, M. E. (2017). Opening the black box: An open‐source release of Maxent. Ecography, 40, 887–893.

[ece311392-bib-0054] Pimenta, M. , de Andrade, A. F. A. , Fernandes, F. H. S. , Amboni, M. P. M. , Almeida, R. S. , Soares, A. H. S. B. , Falcon, G. B. , Raíces, D. S. L. , & De Marco Júnior, P. (2022). One size does not fit all: Priority areas for real world problems. Ecological Modelling, 470, 110013.

[ece311392-bib-0055] Platts, P. J. , Ahrends, A. , Gereau, R. E. , McClean, C. J. , Lovett, J. C. , Marshall, A. R. , Pellikka, P. K. E. , Mulligan, M. , Fanning, E. , & Marchant, R. (2010). Can distribution models help refine inventory‐based estimates of conservation priority? A case study in the eastern arc forests of Tanzania and Kenya. Diversity and Distributions, 16, 628–642.

[ece311392-bib-0056] Prasad, A. M. , Iverson, L. R. , & Liaw, A. (2006). Newer classification and regression tree techniques: Bagging and random forests for ecological prediction. Ecosystems, 9, 181–199.

[ece311392-bib-0112] Projeto Mapbiomas . (2023). Coleção [2.0] da Série Anual de Mapas de Uso e Cobertura da Terra do Brasil, acessado em [2023] através do link: http://amazonia.mapbiomas.org/mapas‐de‐la‐coleccion

[ece311392-bib-0057] Ramírez‐Fráncel, L. A. , García‐Herrera, L. V. , Losada‐Prado, S. , Reinoso‐Flórez, G. , Sánchez‐Hernández, A. , Estrada‐Villegas, S. , Lim, B. K. , & Guevara, G. (2022). Bats and their vital ecosystem services: A global review. Integrative Zoology, 17, 2–23. 34003577 10.1111/1749-4877.12552

[ece311392-bib-0058] Raxworthy, C. J. , Martinez‐Meyer, E. , Horning, N. , Nussbaum, R. A. , Schneider, G. E. , Ortega‐Huerta, M. A. , & Townsend Peterson, A. (2003). Predicting distributions of known and unknown reptile species in Madagascar. Nature, 426, 837–841. 14685238 10.1038/nature02205

[ece311392-bib-0059] Razgour, O. , Hanmer, J. , & Jones, G. (2011). Using multi‐scale modelling to predict habitat suitability for species of conservation concern: The grey long‐eared bat as a case study. Biological Conservation, 144, 2922–2930.

[ece311392-bib-0113] R Core Team . (2010). R: A language and environment for statistical computing. R Foundation for Statistical Computing. https://www.R‐project.org/

[ece311392-bib-0060] Schnitzler, H. U. , & Kalko, E. K. (2001). Echolocation by insect‐eating bats: We define four distinct functional groups of bats and find differences in signal structure that correlate with the typical echolocation tasks faced by each group. Bioscience, 51(7), 557–569.

[ece311392-bib-0114] Shabani, F. , Kumar, L. , & Ahmadi, M. (2018). Assessing accuracy methods of species distributionmodels: AUC, Specificity, Sensitivity and the True Skill Statistic. Global Journal of Human‐Social Science, 18, 6–18.

[ece311392-bib-0061] Silva, D. C. , Vieira, T. B. , da Silva, J. M. , & de Cassia Faria, K. (2018). Biogeography and priority areas for the conservation of bats in the Brazilian Cerrado. Biodiversity and Conservation, 27(4), 815–828.

[ece311392-bib-0062] Silva, M. E. S. , Pereira, G. , & da Rocha, R. P. (2016). Local and remote climatic impacts due to land use degradation in the Amazon “arc of deforestation”. Theoretical and Applied Climatology, 125(3–4), 609–623.

[ece311392-bib-0063] Simmons, N. B. , & Cirranello, A. L. (2023). Bat Species of the World: A taxonomic and geographic reference . https://batnames.org

[ece311392-bib-0064] Sipinski, E. A. B. , & Reis, N. R. (1995). Dado secológicos dos quirópteros da Reserva de Volta Velha, Itapoá, Santa Catarina, Brasil. Revista Brasileira de Zoologia, 12, 519–528.

[ece311392-bib-0065] Smith, T. D. , Santana, S. E. , & Eiting, T. P. (2023). Ecomorphology and sensory biology of bats. The Anatomical Record, 306(11), 2660–2669. 37656052 10.1002/ar.25314

[ece311392-bib-0066] Soberón, J. , & Nakamura, M. (2009). Niches and distributional areas: Concepts, methods, and assumptions. Proceedings of the National Academy of Sciences of the United States of America, 106, 19644–19650. 19805041 10.1073/pnas.0901637106 PMC2780935

[ece311392-bib-0067] Sousa‐Baena, M. S. , Garcia, L. C. , & Peterson, A. T. (2014). Knowledge behind conservation status decisions: Data basis for “data deficient” Brazilian plant species. Biological Conservation, 173, 80–89.

[ece311392-bib-0068] Straube, F. C. , & Bianconi, G. V. (2002). Sobre a grandeza e a unidade utilizada para estimar esforço de captura com utilização de redes‐de‐neblina. Chiroptera Neotropical, 8(1–2), 150–152.

[ece311392-bib-0069] Suripto, B. (2021). Economic contribution of fruit bats (family Pteropodidae) through durian fruit production in the agroecosystem in Java Island . Proceedings of the 7th international conference on research, implementation, and education of mathematics and sciences (ICRIEMS 2020), 528, 8–15.

[ece311392-bib-0115] Velazco, S. J. E. , Villalobos, F. , Galvão, F. , & De Marco Júnior, P. (2019). A dark scenario for Cerrado plant species: Effects of future climate, land use and protected areas ineffectiveness. Diversity and Distributions, 25, 660–673. 10.1111/ddi.12886

[ece311392-bib-0070] Vieira, T. B. , da Silva, L. C. N. , Aguiar, L. M. , Oprea, M. , Mendes, P. , & Ditchfield, A. D. (2021). Bat species composition associated with restinga lagoons from the Paulo césar vinha state park, Espírito Santo, Brazil. Papéis Avulsos de Zoologia., 61, e20216132.

[ece311392-bib-0071] Voigt, C. C. , & Kingston, T. (2016). Bats in the Anthropocene: Conservation of bats in a changing world (p. 606). Springer Nature.

[ece311392-bib-0072] Zortéa, M. , Ribeiro, M. C. S. , da Mata, P. S. , & Bonvicino, C. R. (2023). Morphological and molecular evidence of the occurrence of *Artibeus amplus* (Chiroptera: Phyllostomidae) in Brazil. Zoologia (Curitiba), 40, e22058.

