# Supplementary figures and images for: Some bats are here: Reducing the Wallacean shortfall of bats in the amazon
Source: Ecol Evol. 2024 Jun 4;14(6):e11392. doi: 10.1002/ece3.11392 (PMC11150423; doi:10.1002/ece3.11392)

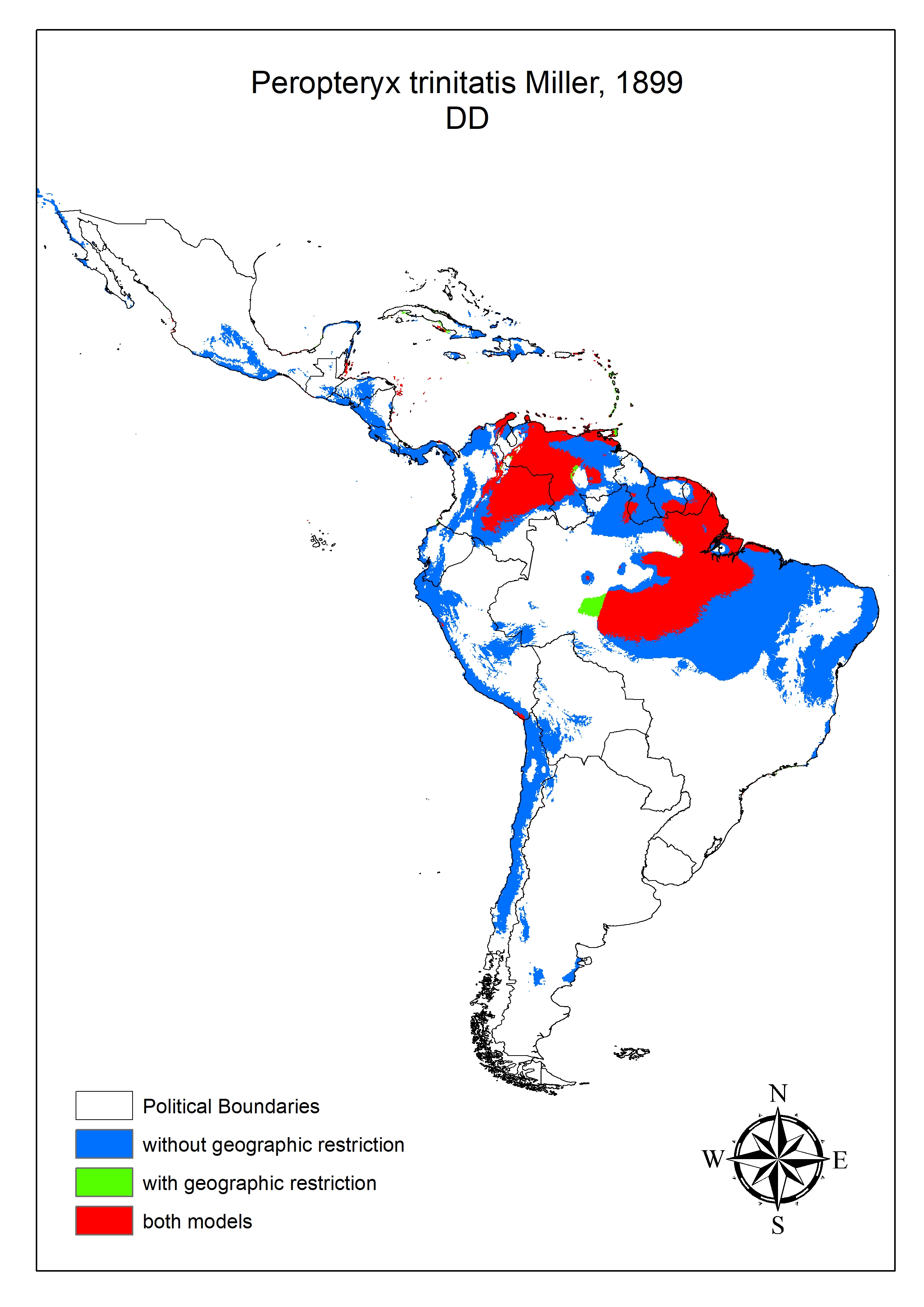

Supplement: Supplementary file 3 — Figure S1. [file ECE3-14-e11392-s016.jpg]

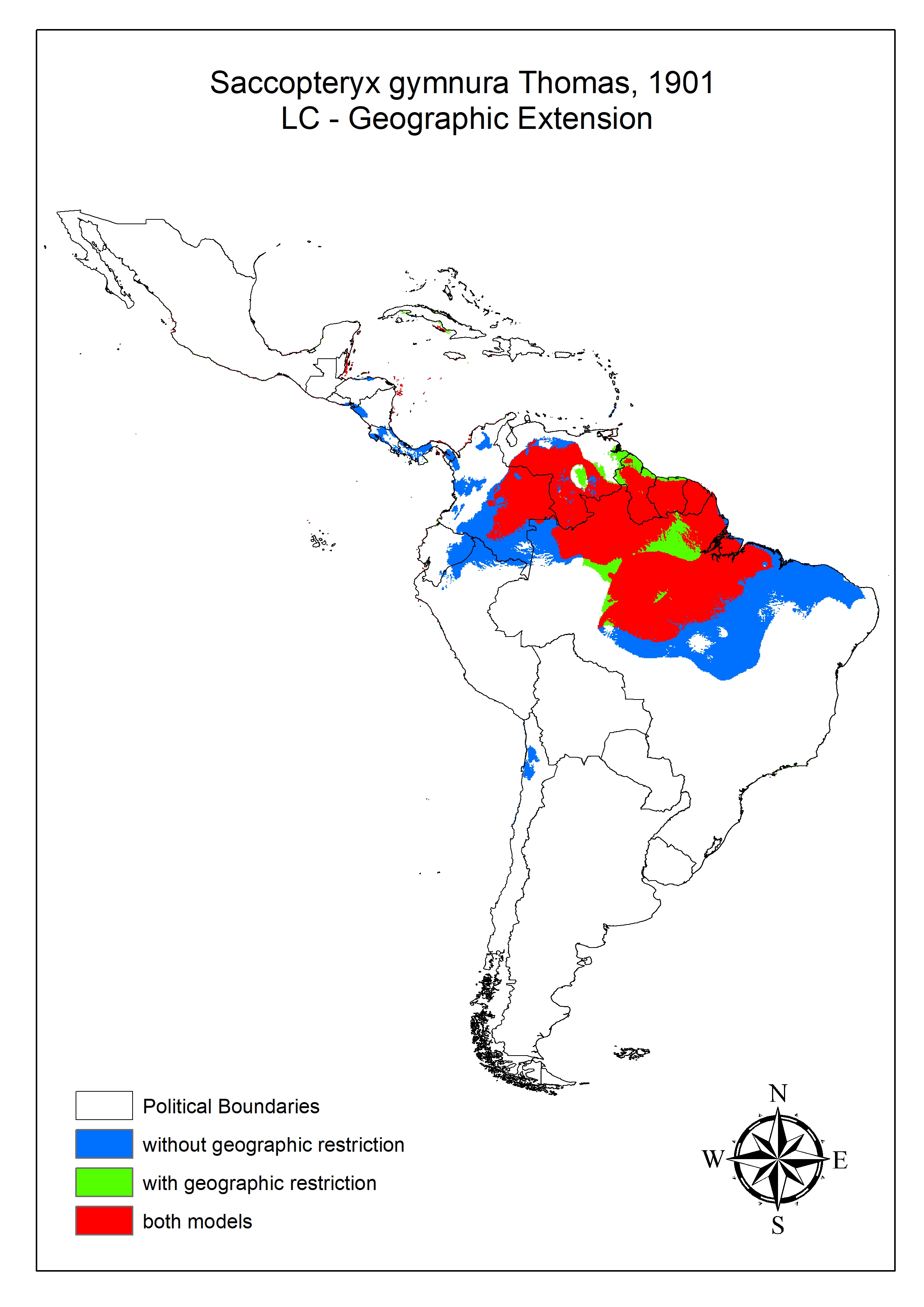

Supplement: Supplementary file 4 — Figure S2. [file ECE3-14-e11392-s019.jpg]

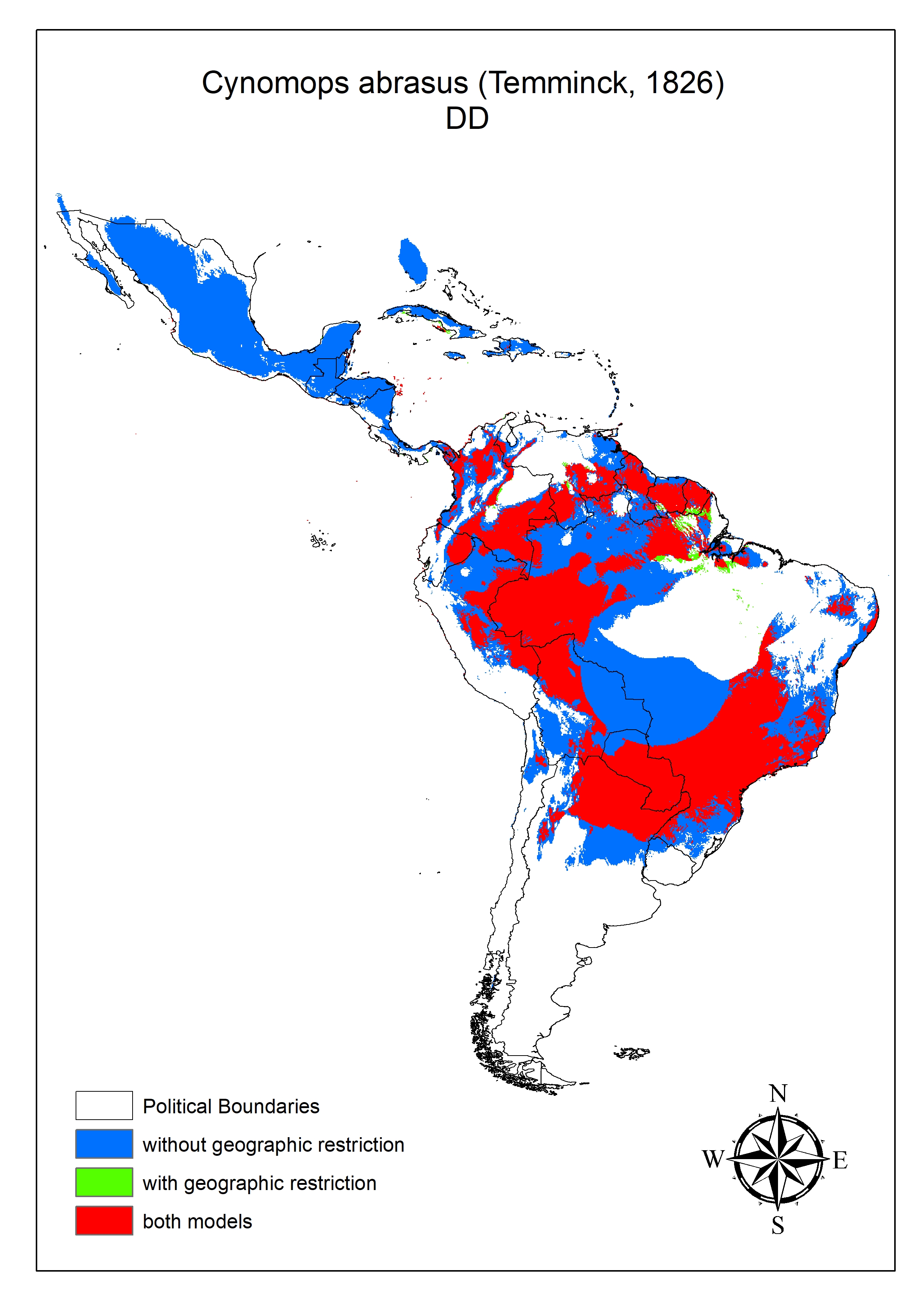

Supplement: Supplementary file 5 — Figure S3. [file ECE3-14-e11392-s002.jpg]

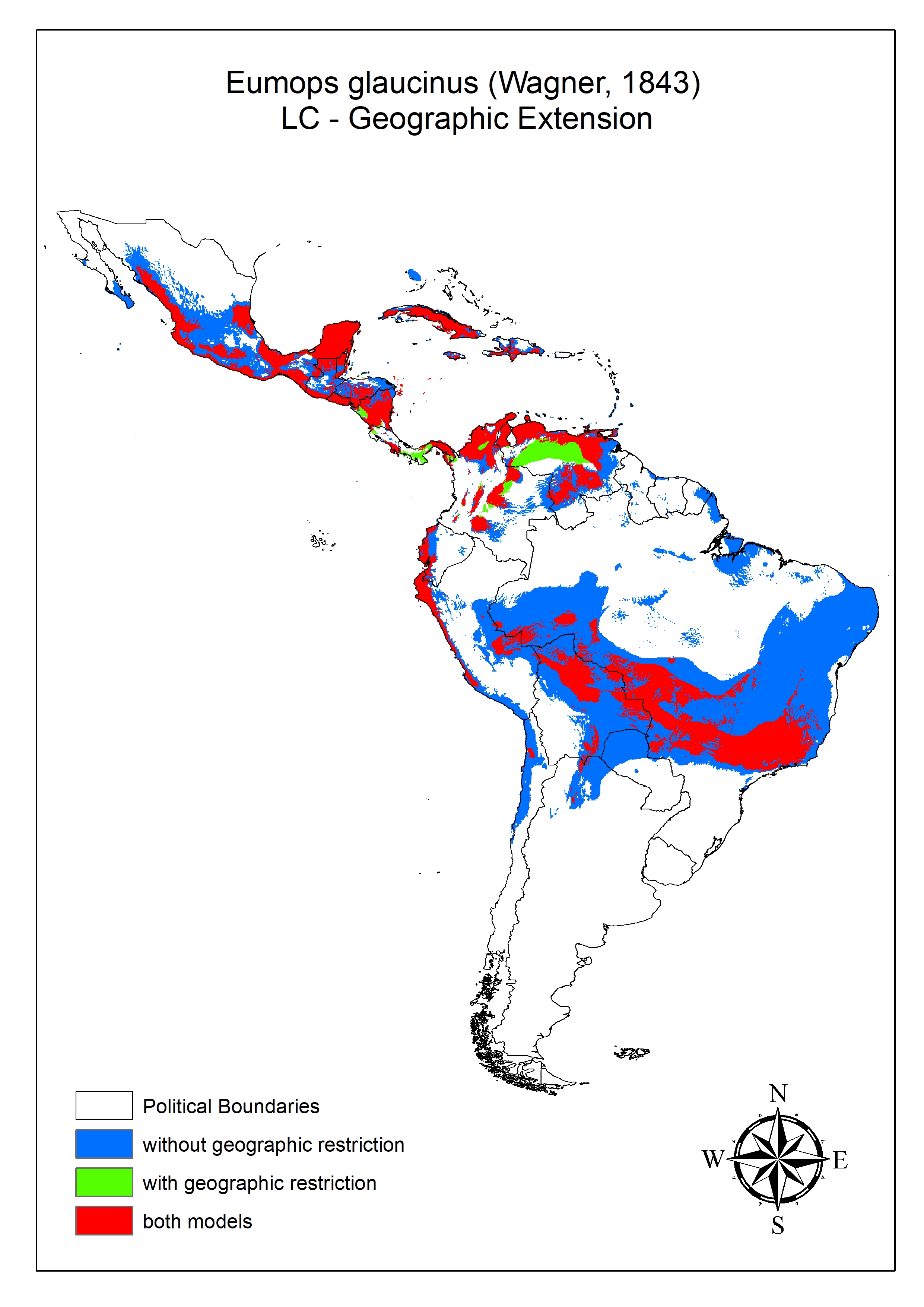

Supplement: Supplementary file 6 — Figure S4. [file ECE3-14-e11392-s021.jpg]

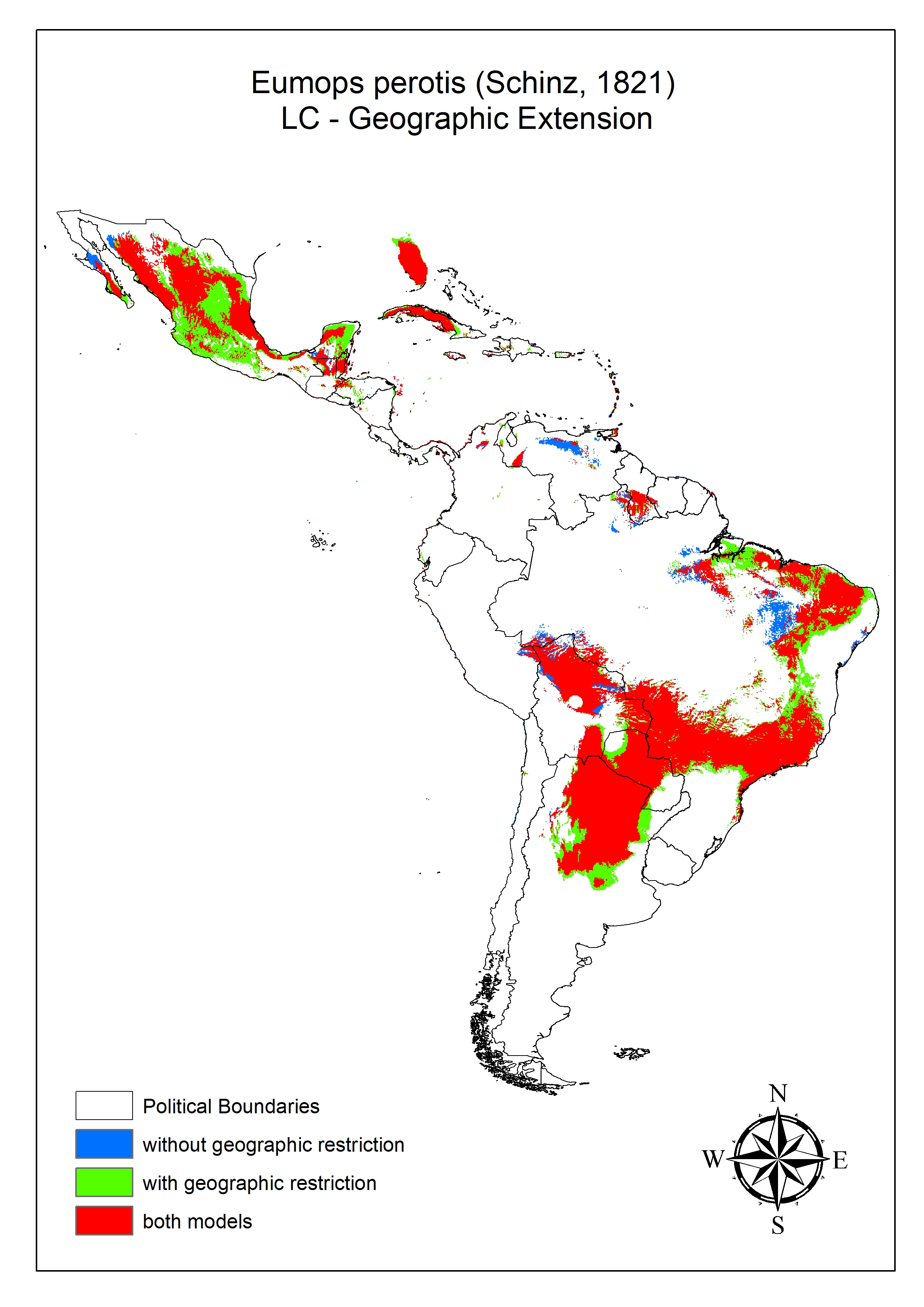

Supplement: Supplementary file 7 — Figure S5. [file ECE3-14-e11392-s011.jpg]

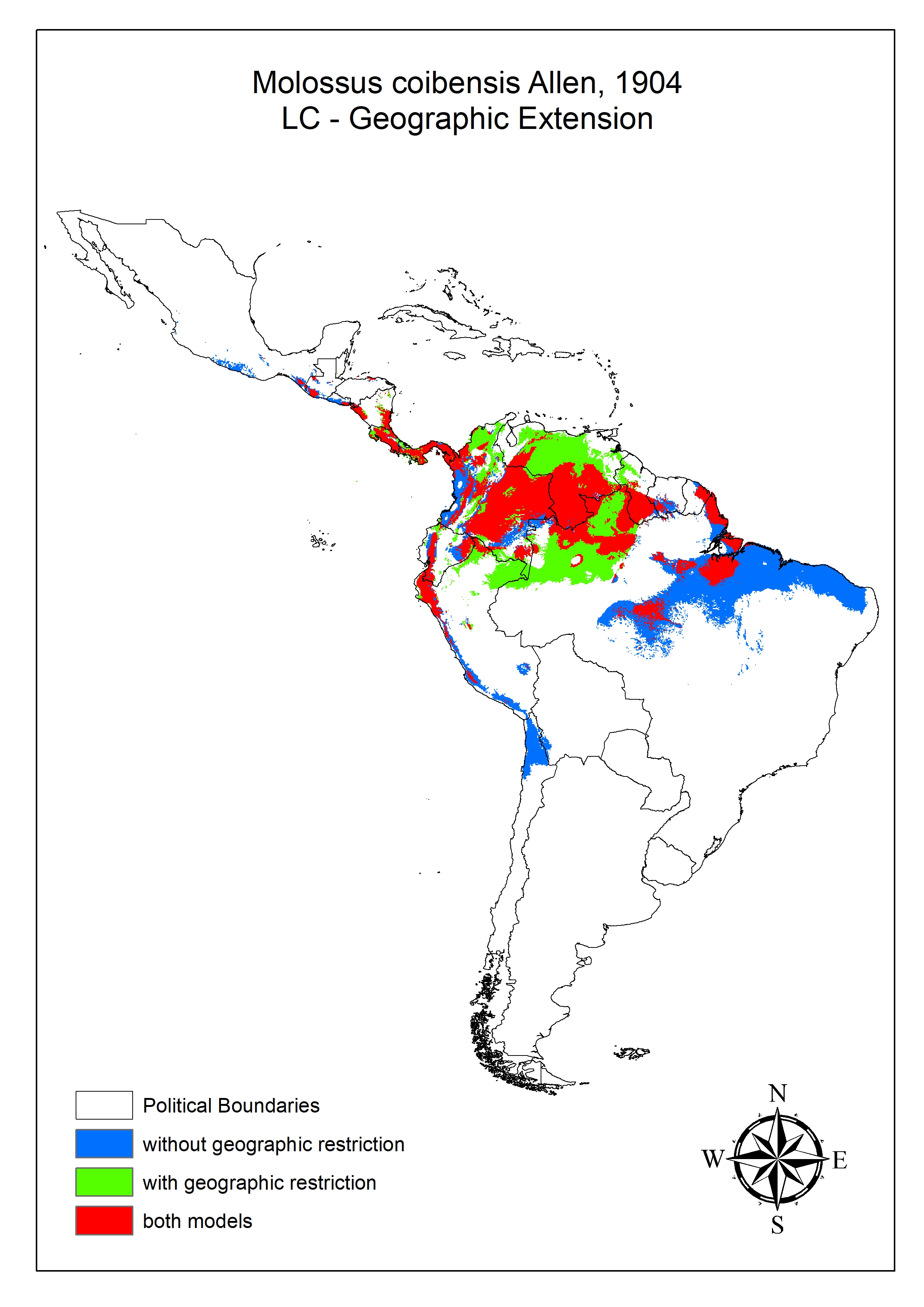

Supplement: Supplementary file 8 — Figure S6. [file ECE3-14-e11392-s008.jpg]

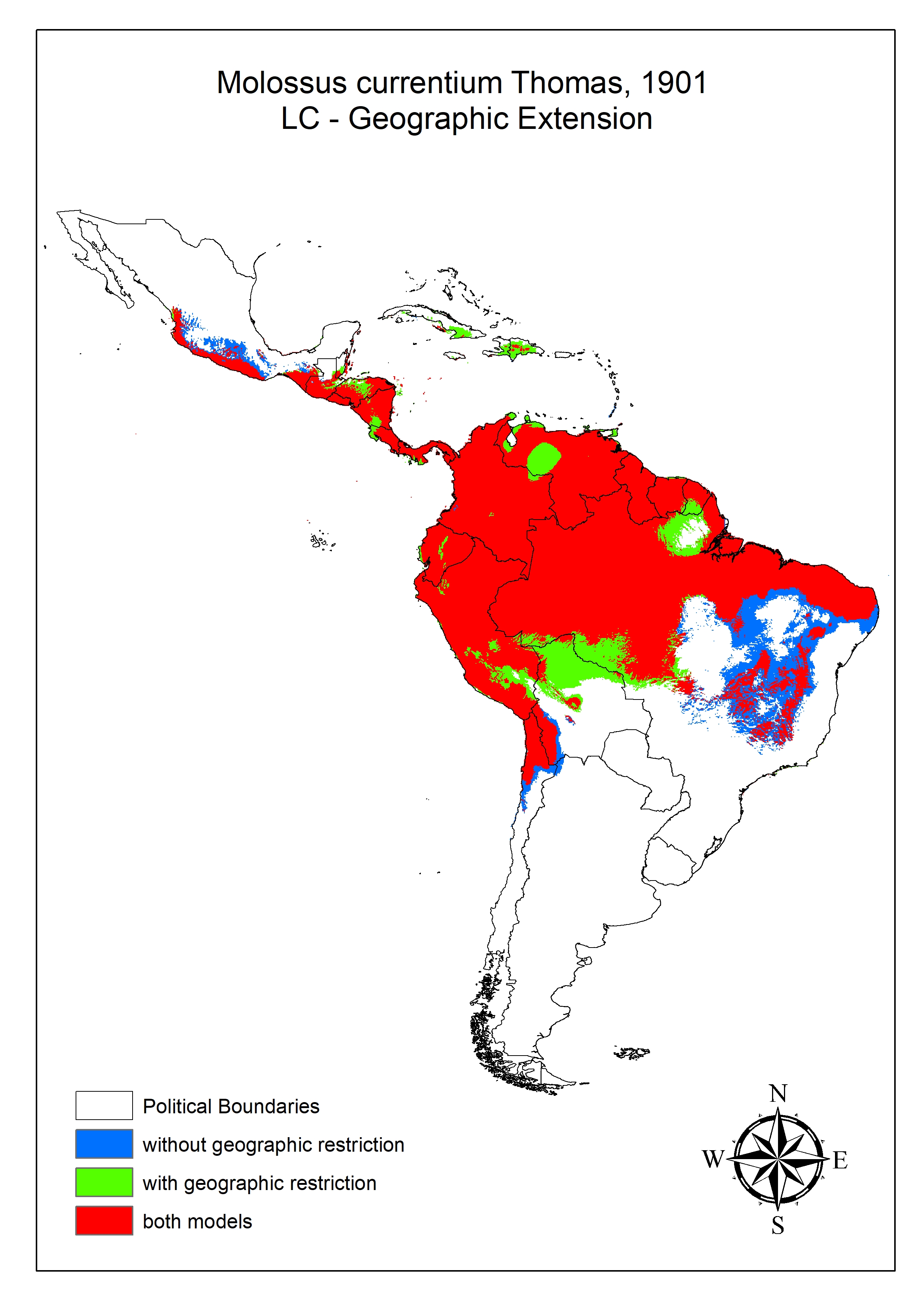

Supplement: Supplementary file 9 — Figure S7. [file ECE3-14-e11392-s020.jpg]

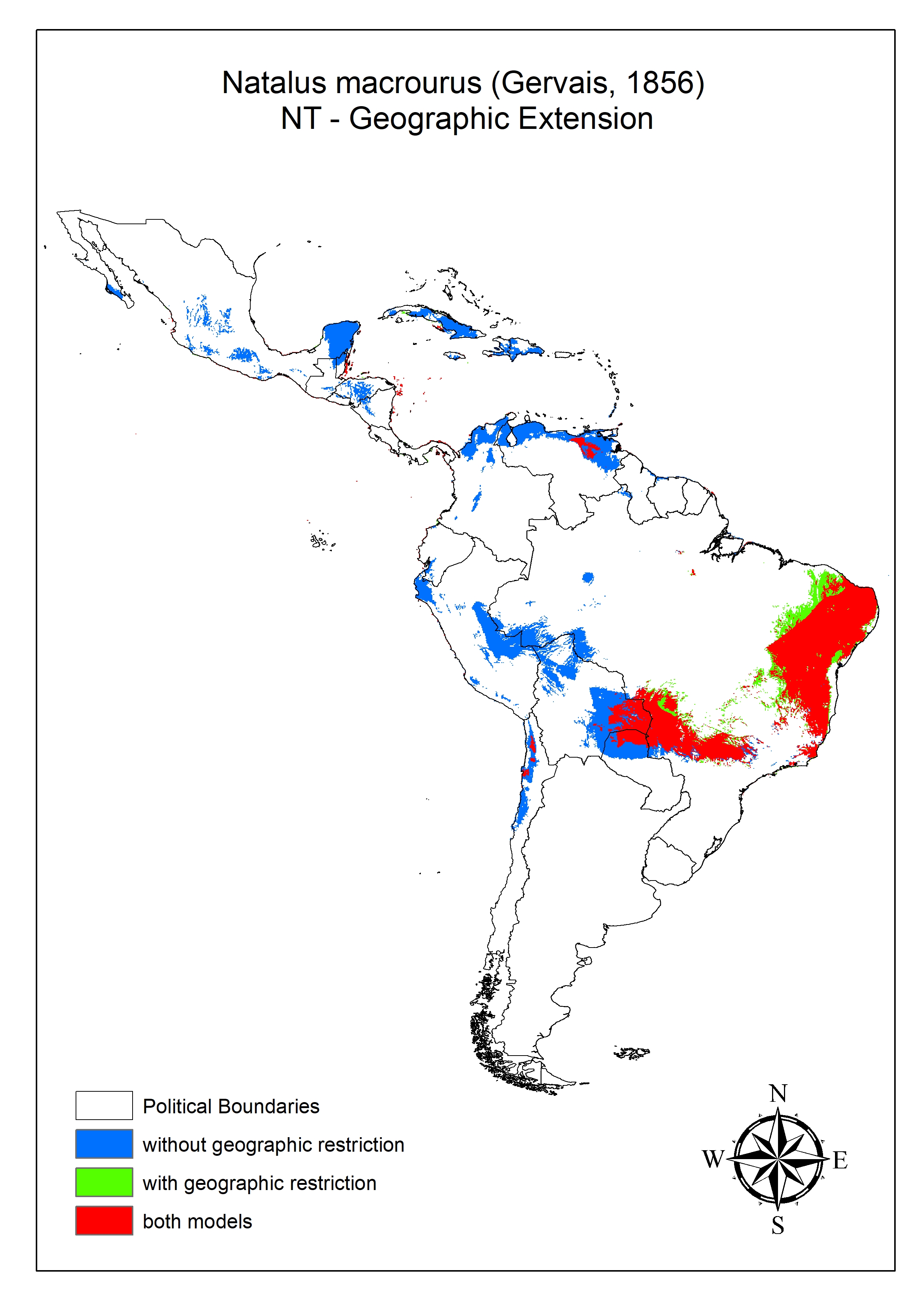

Supplement: Supplementary file 10 — Figure S8. [file ECE3-14-e11392-s005.jpg]

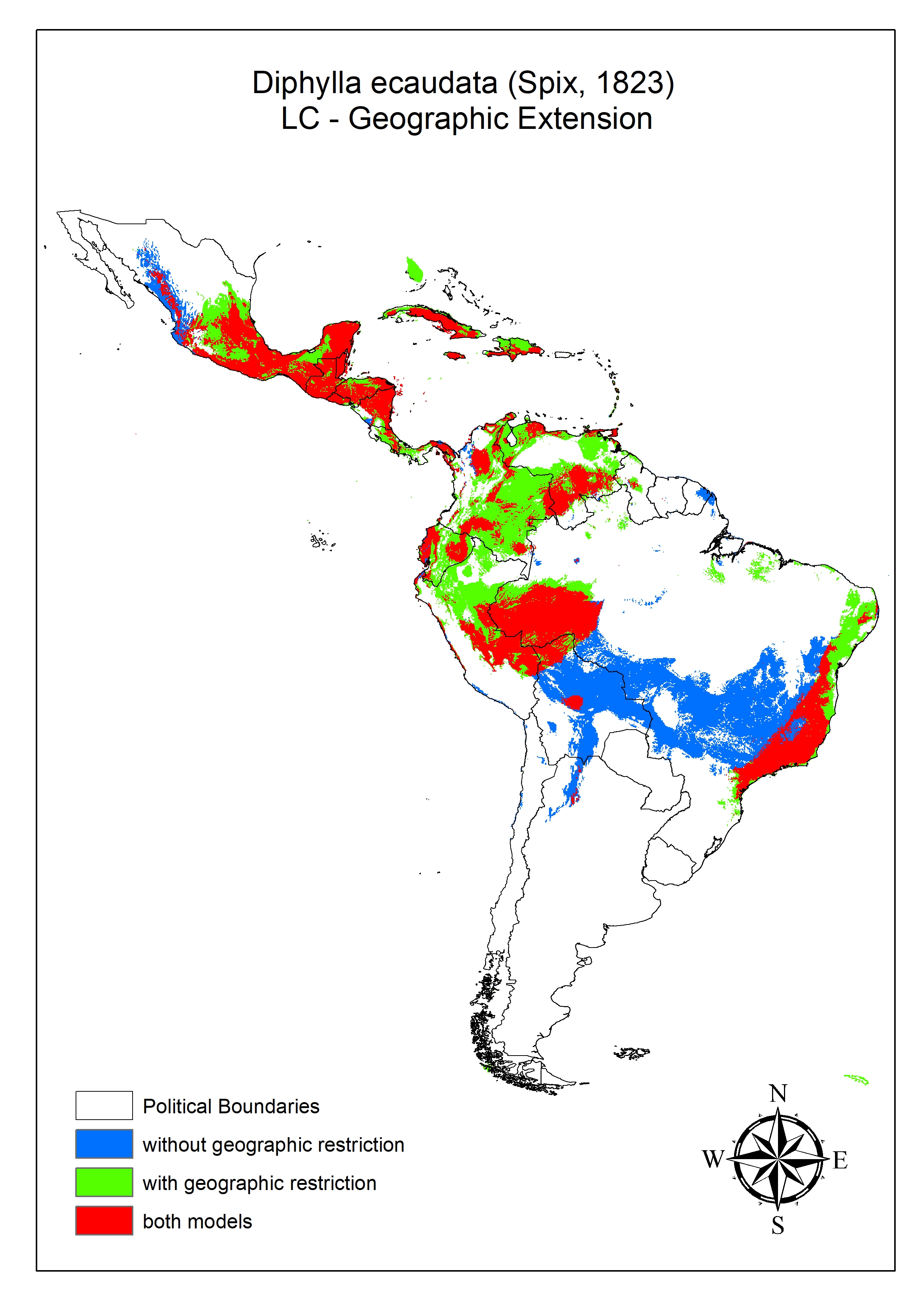

Supplement: Supplementary file 11 — Figure S9. [file ECE3-14-e11392-s024.jpg]

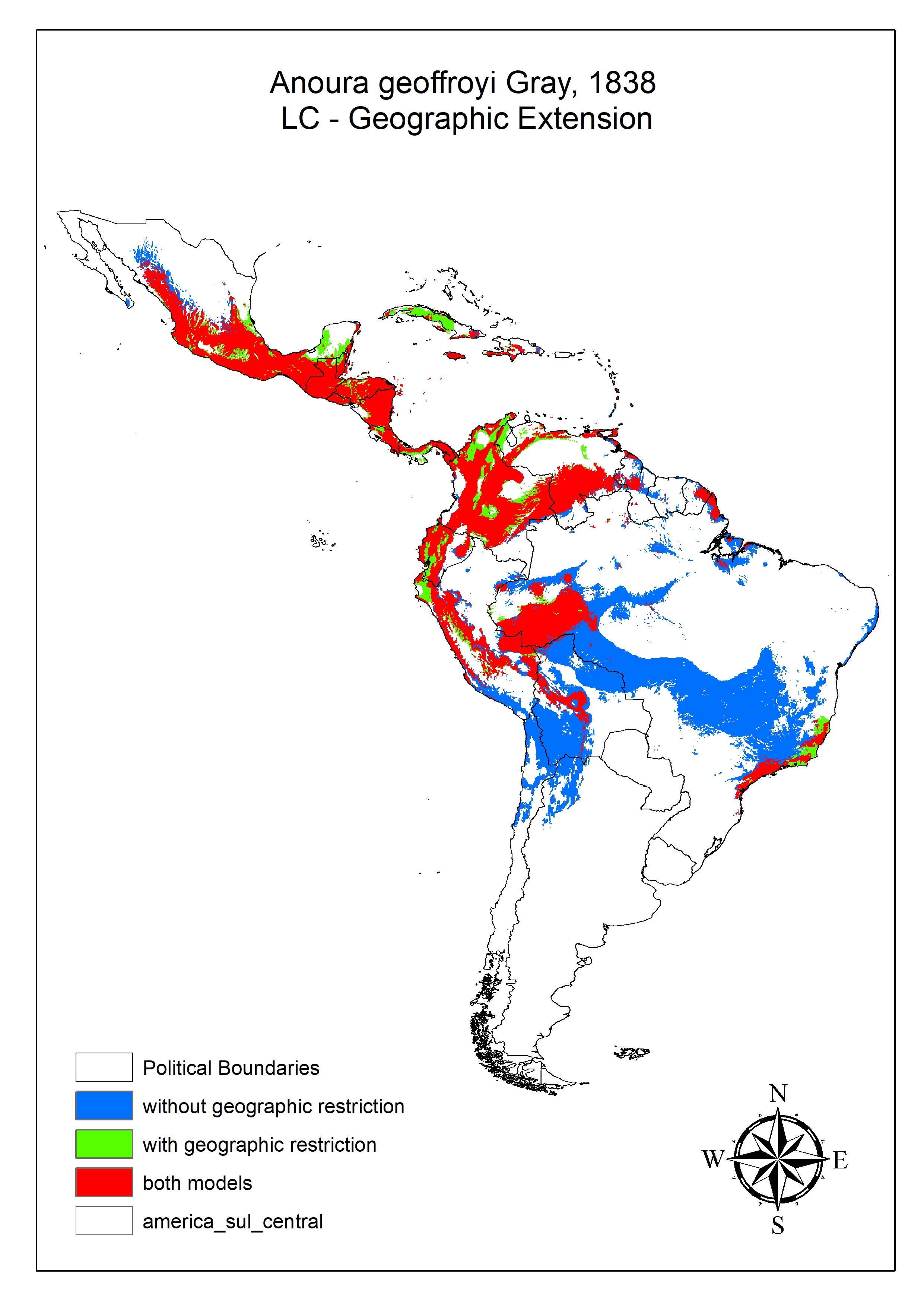

Supplement: Supplementary file 12 — Figure S10. [file ECE3-14-e11392-s014.jpg]

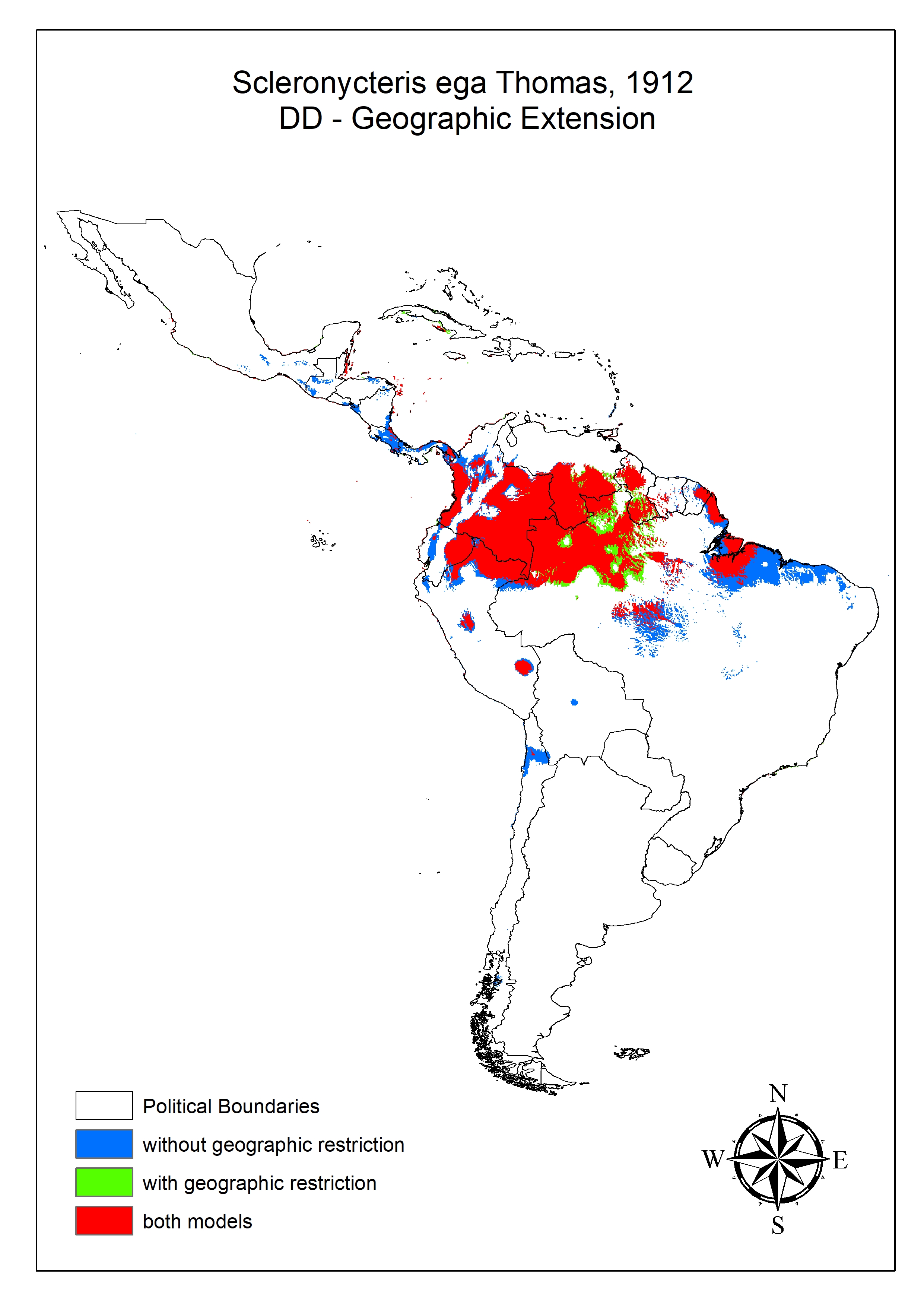

Supplement: Supplementary file 13 — Figure S11. [file ECE3-14-e11392-s001.jpg]

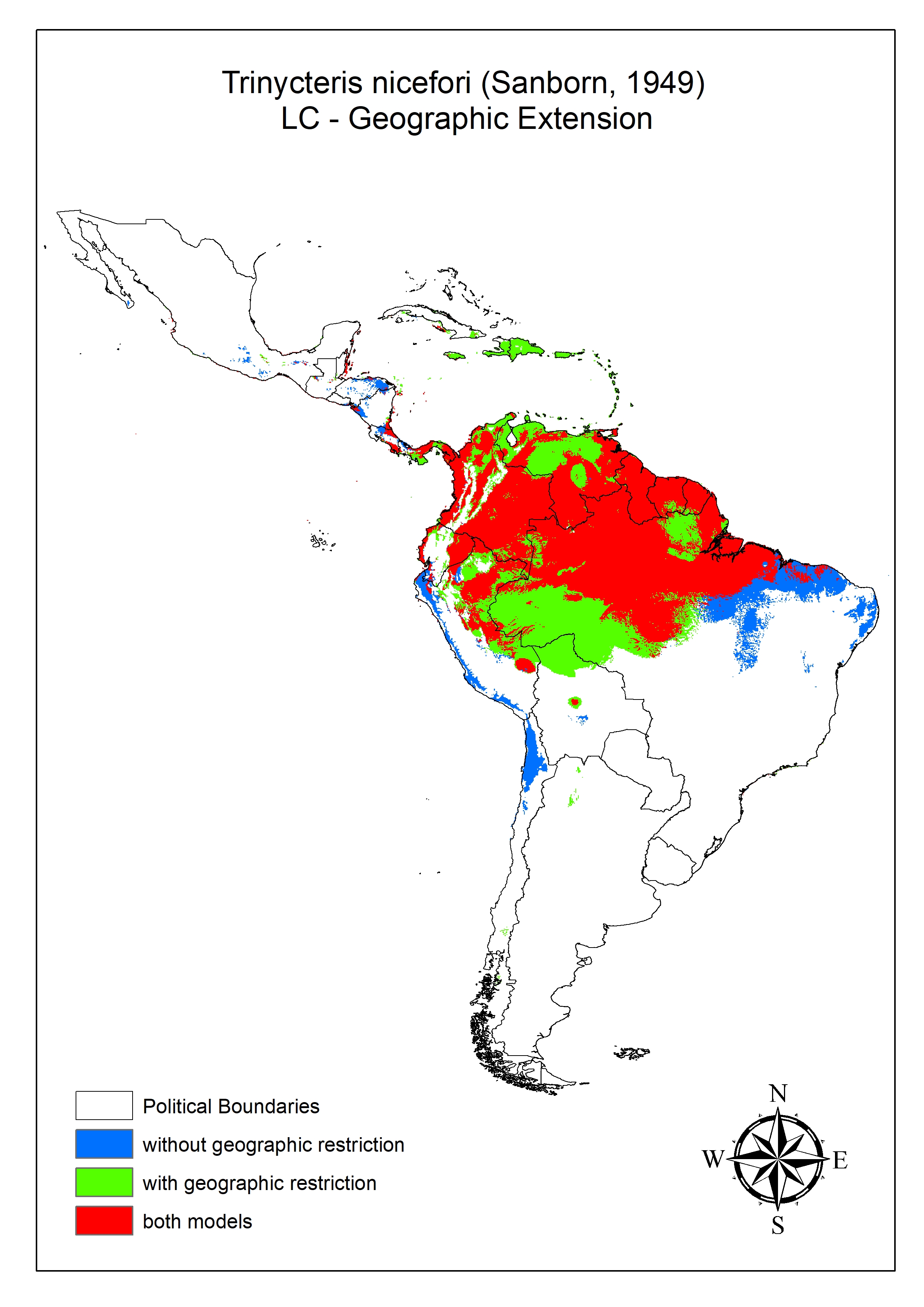

Supplement: Supplementary file 14 — Figure S12. [file ECE3-14-e11392-s022.jpg]

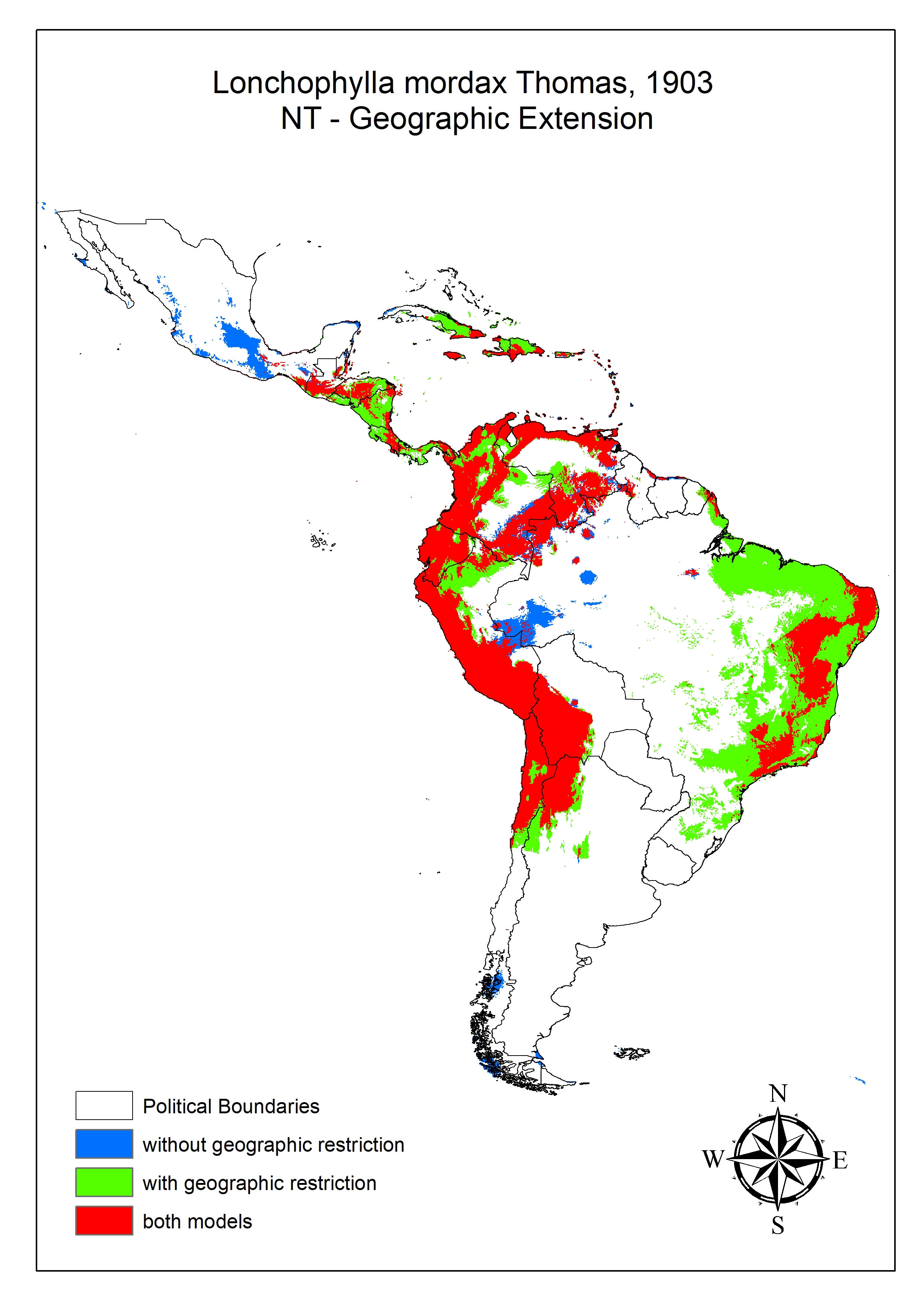

Supplement: Supplementary file 15 — Figure S13. [file ECE3-14-e11392-s007.jpg]

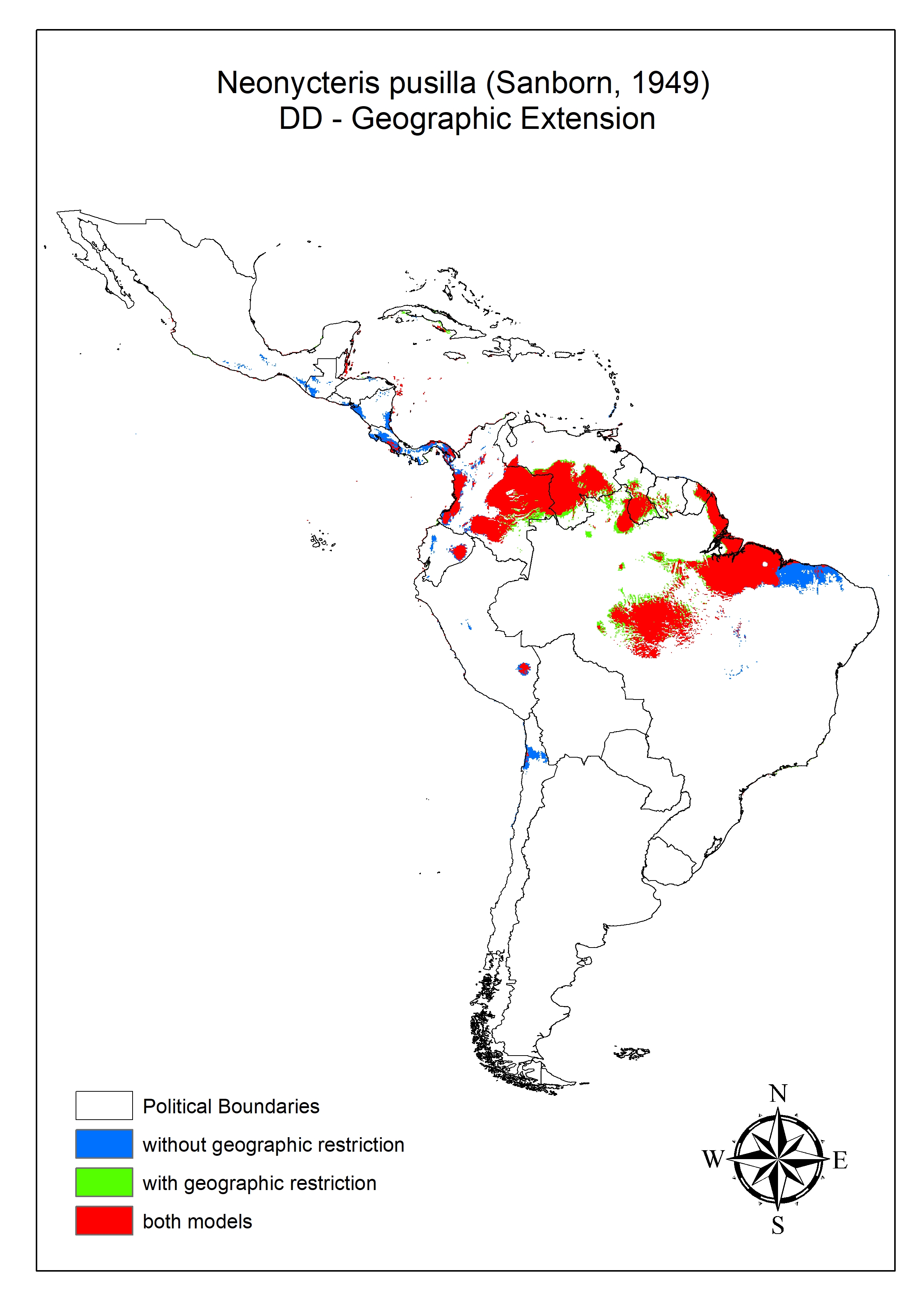

Supplement: Supplementary file 16 — Figure S14. [file ECE3-14-e11392-s025.jpg]

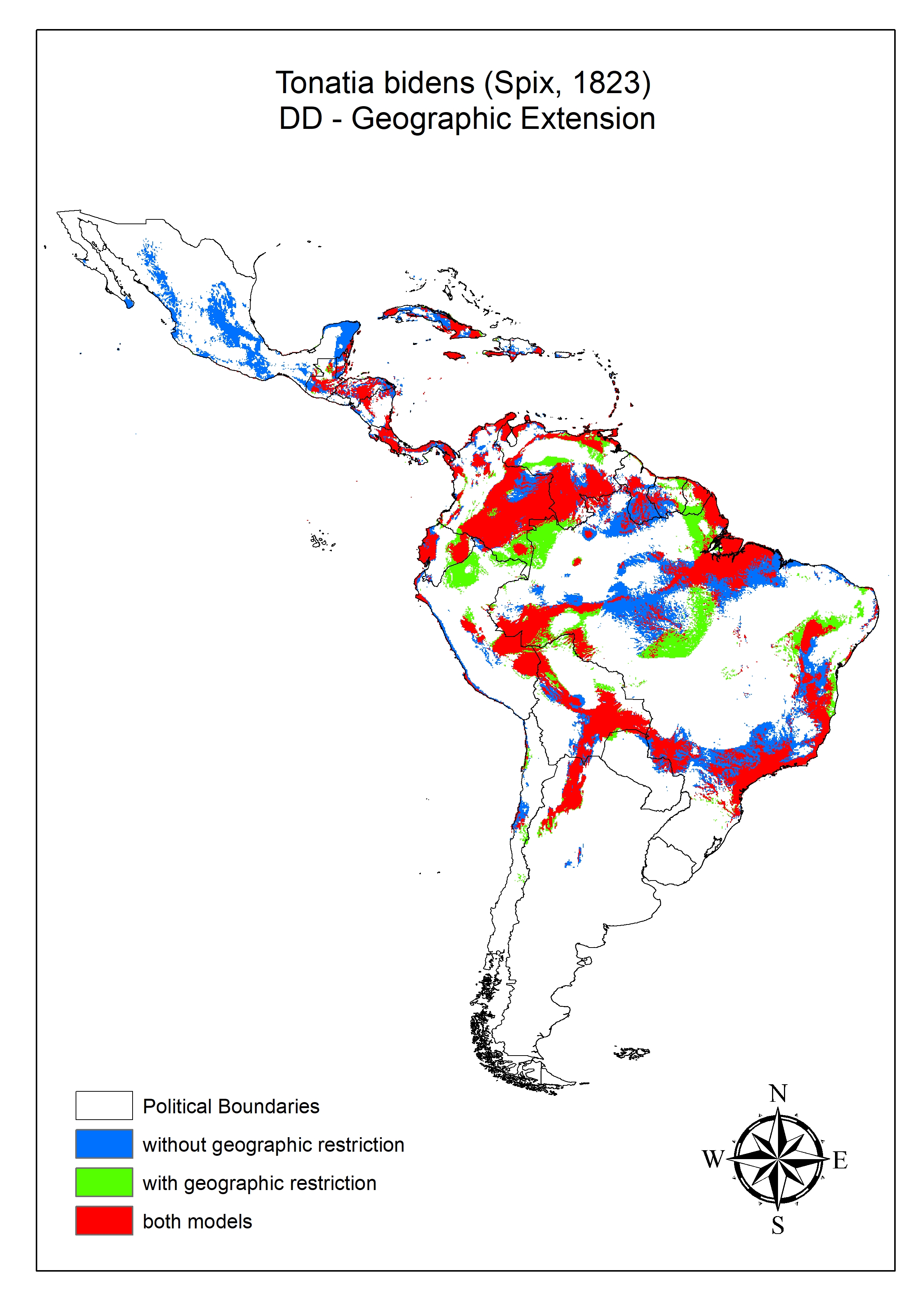

Supplement: Supplementary file 17 — Figure S15. [file ECE3-14-e11392-s012.jpg]

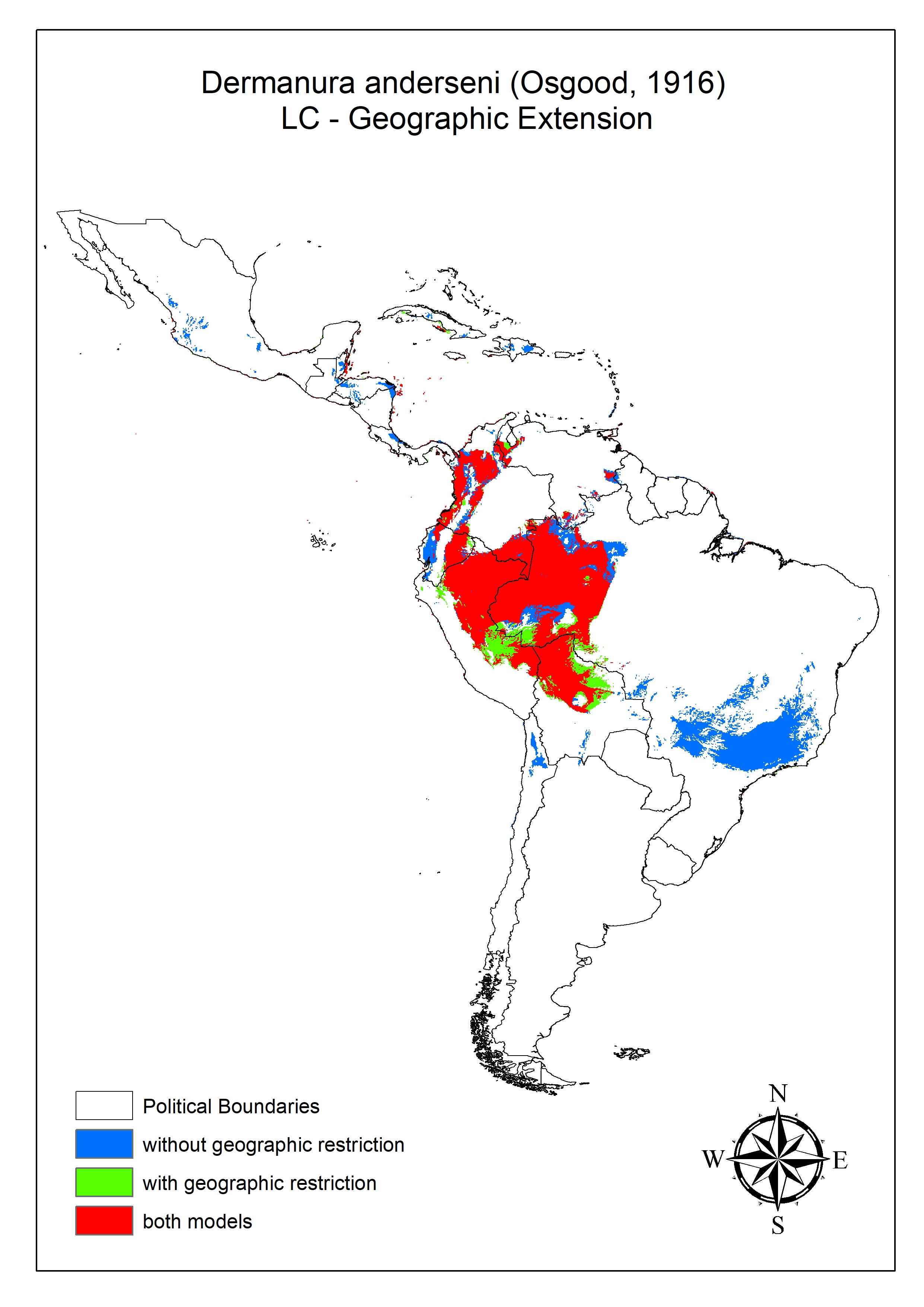

Supplement: Supplementary file 18 — Figure S16. [file ECE3-14-e11392-s009.jpg]

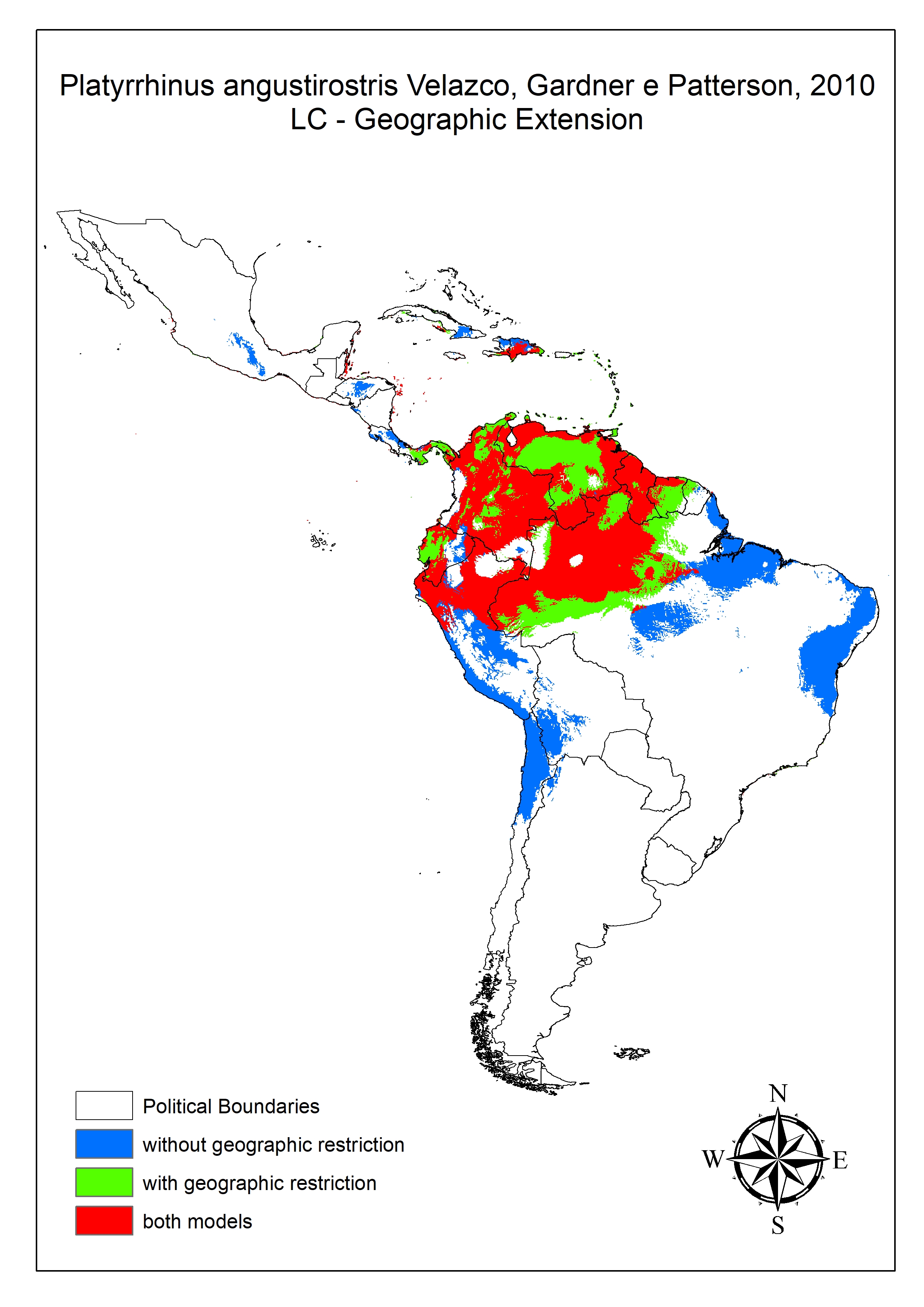

Supplement: Supplementary file 19 — Figure S17. [file ECE3-14-e11392-s023.jpg]

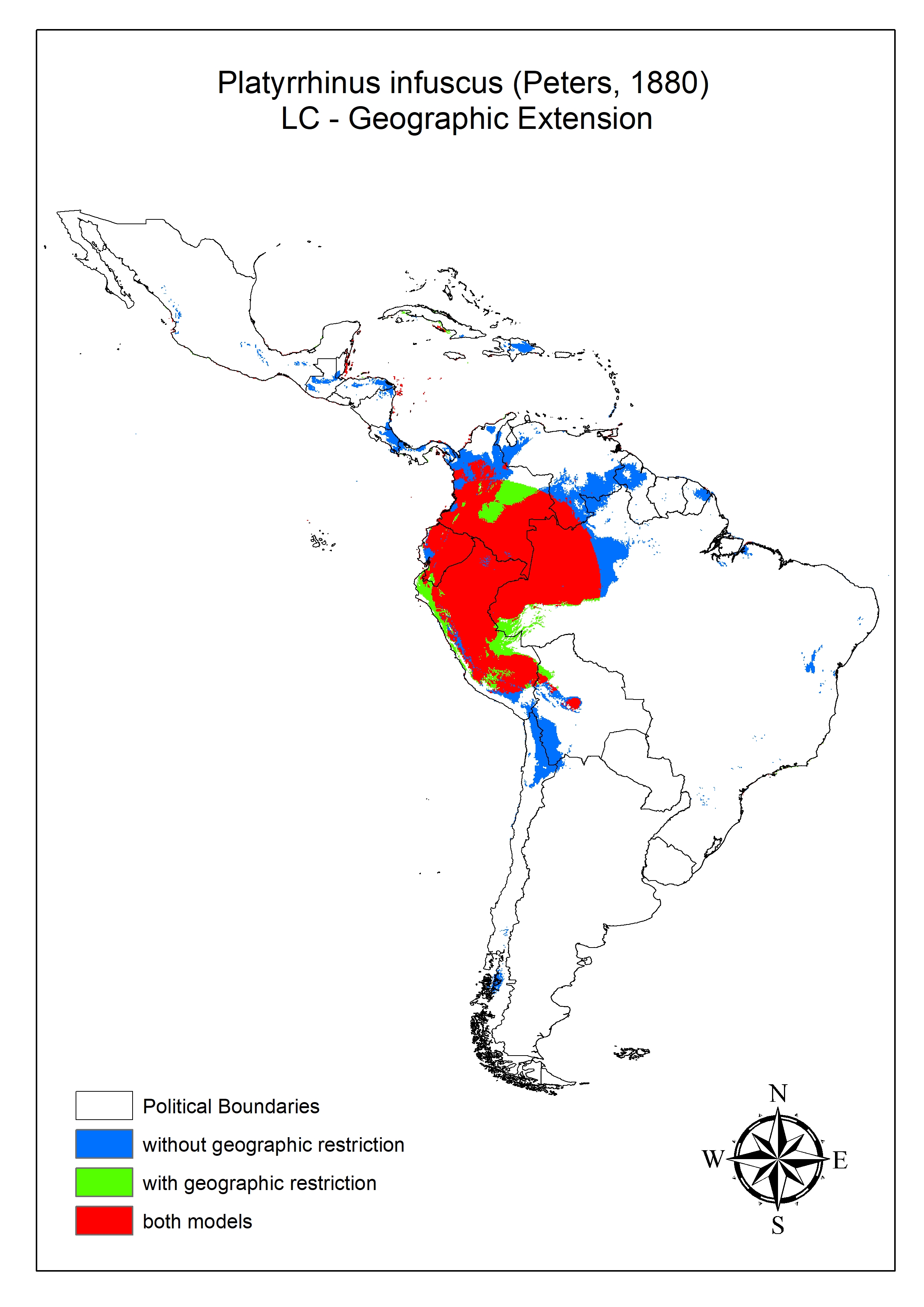

Supplement: Supplementary file 20 — Figure S18. [file ECE3-14-e11392-s004.jpg]

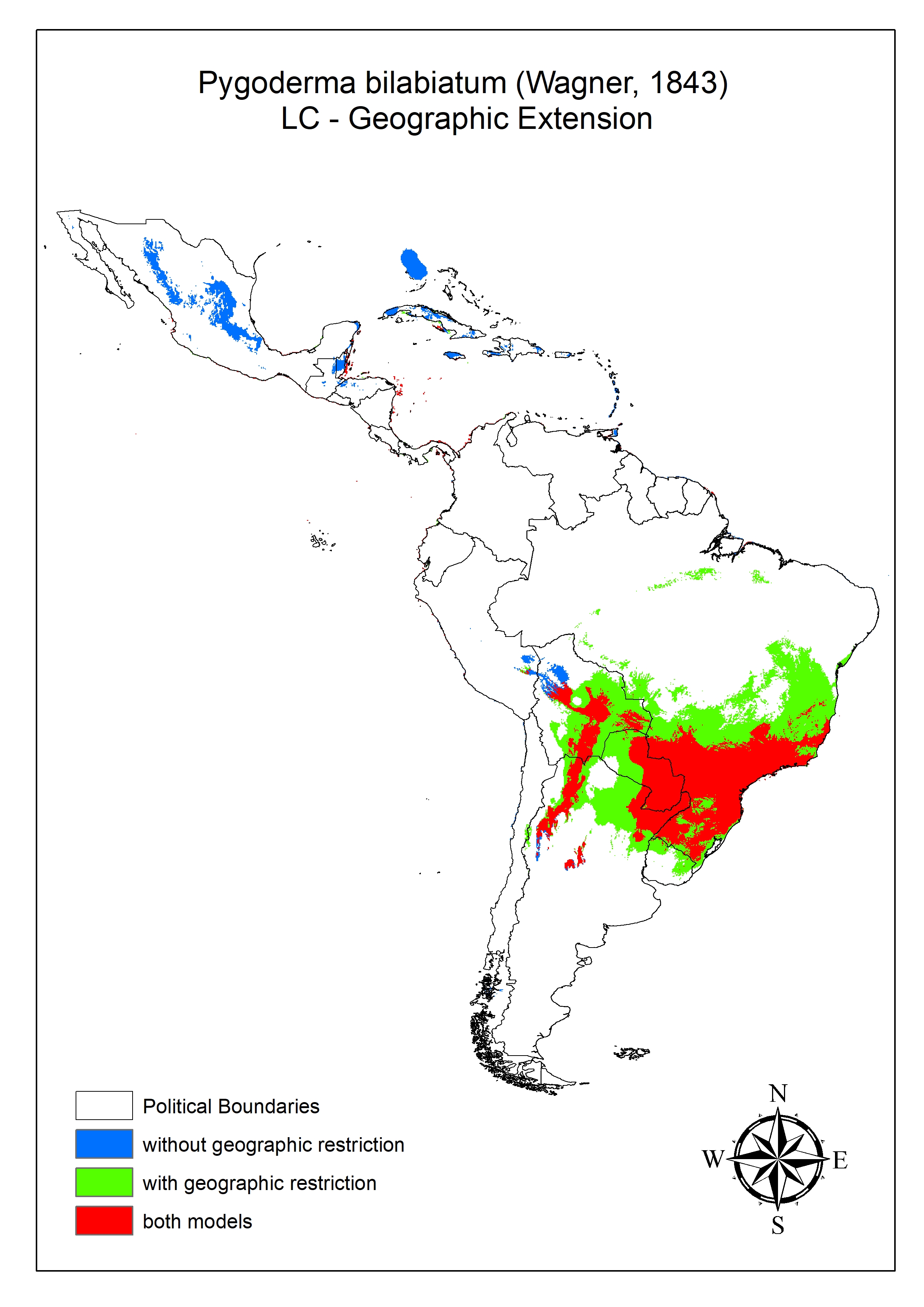

Supplement: Supplementary file 21 — Figure S19. [file ECE3-14-e11392-s018.jpg]

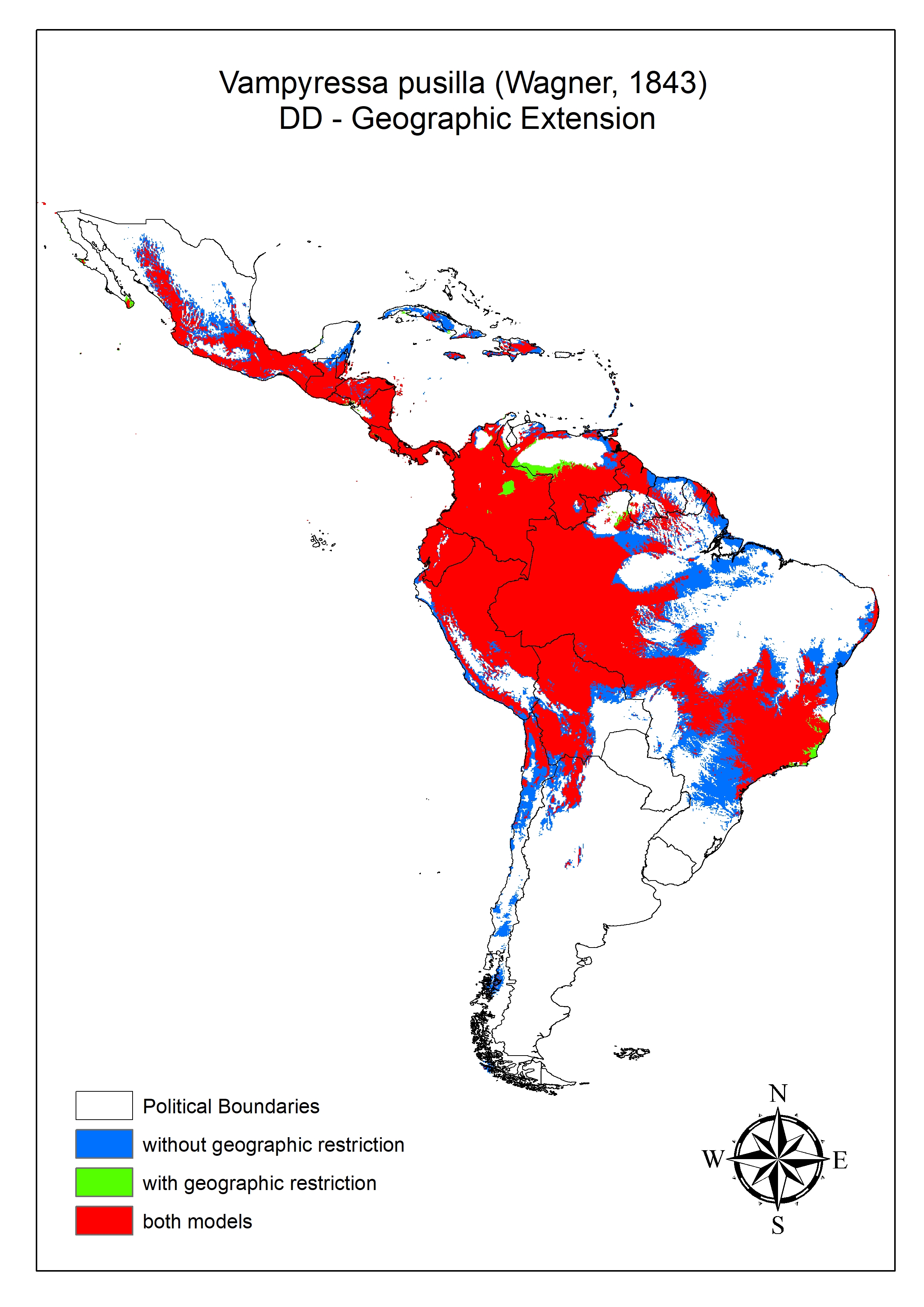

Supplement: Supplementary file 22 — Figure S20. [file ECE3-14-e11392-s003.jpg]

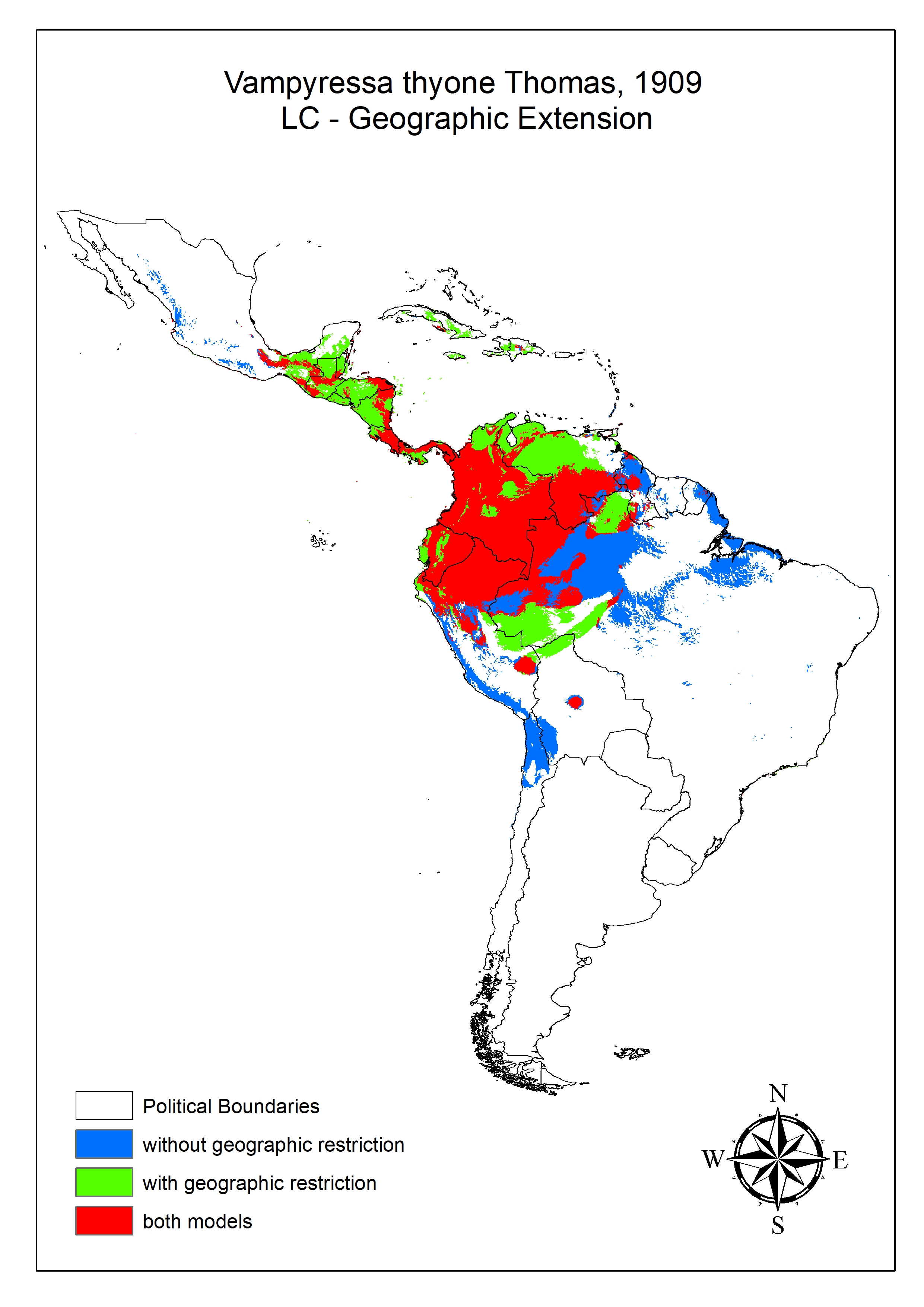

Supplement: Supplementary file 23 — Figure S21. [file ECE3-14-e11392-s017.jpg]

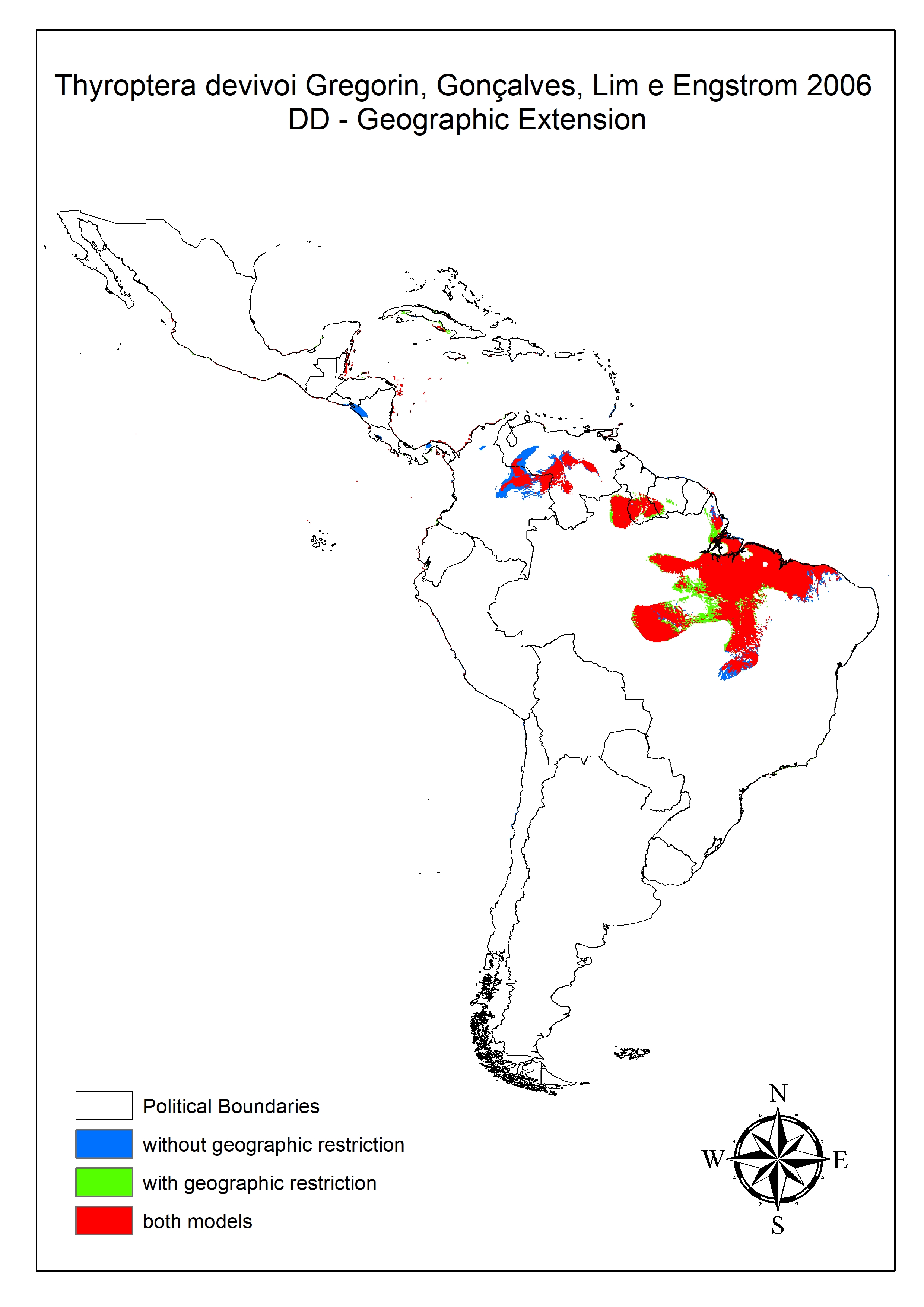

Supplement: Supplementary file 24 — Figure S22. [file ECE3-14-e11392-s010.jpg]

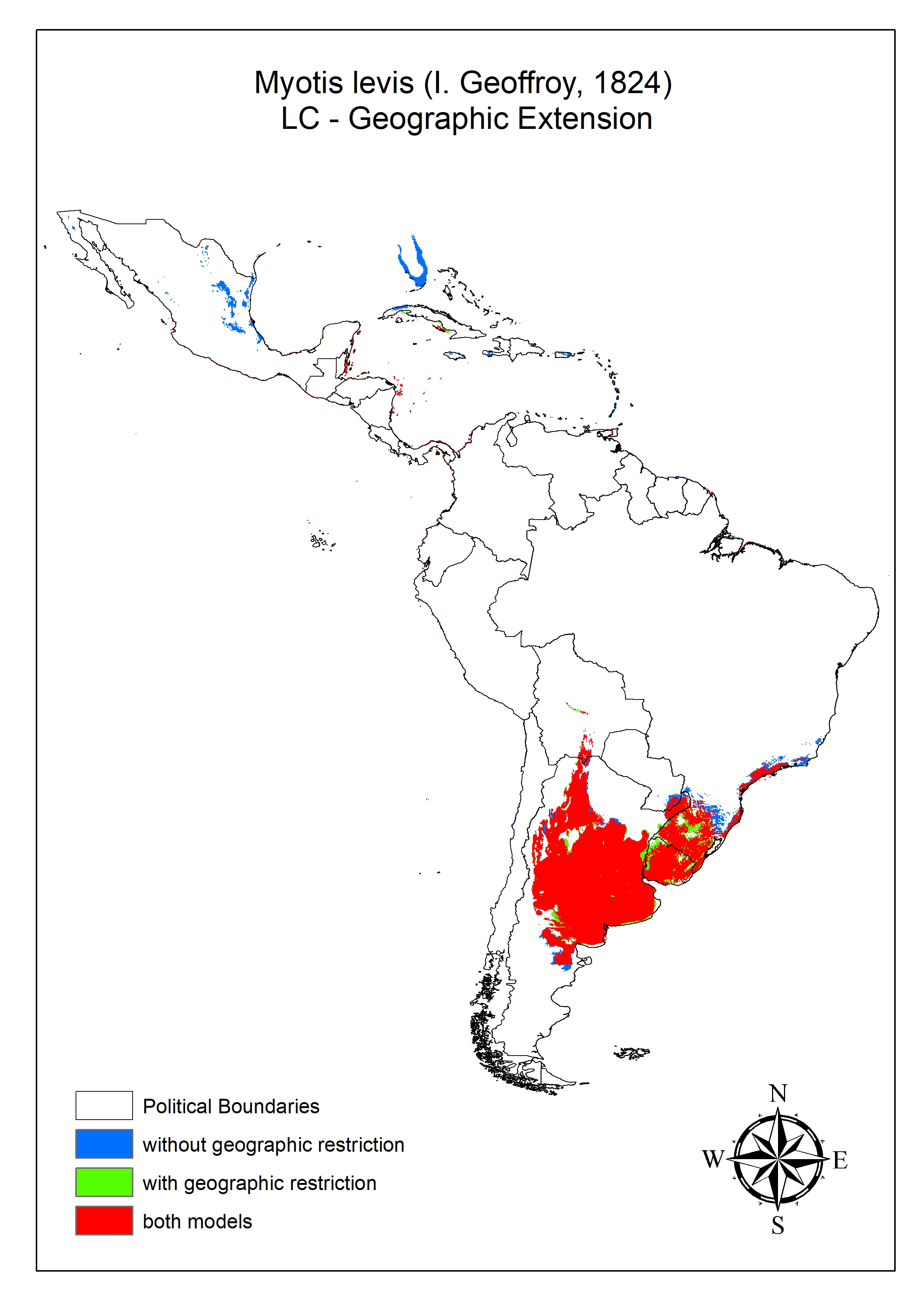

Supplement: Supplementary file 25 — Figure S23. [file ECE3-14-e11392-s026.jpg]

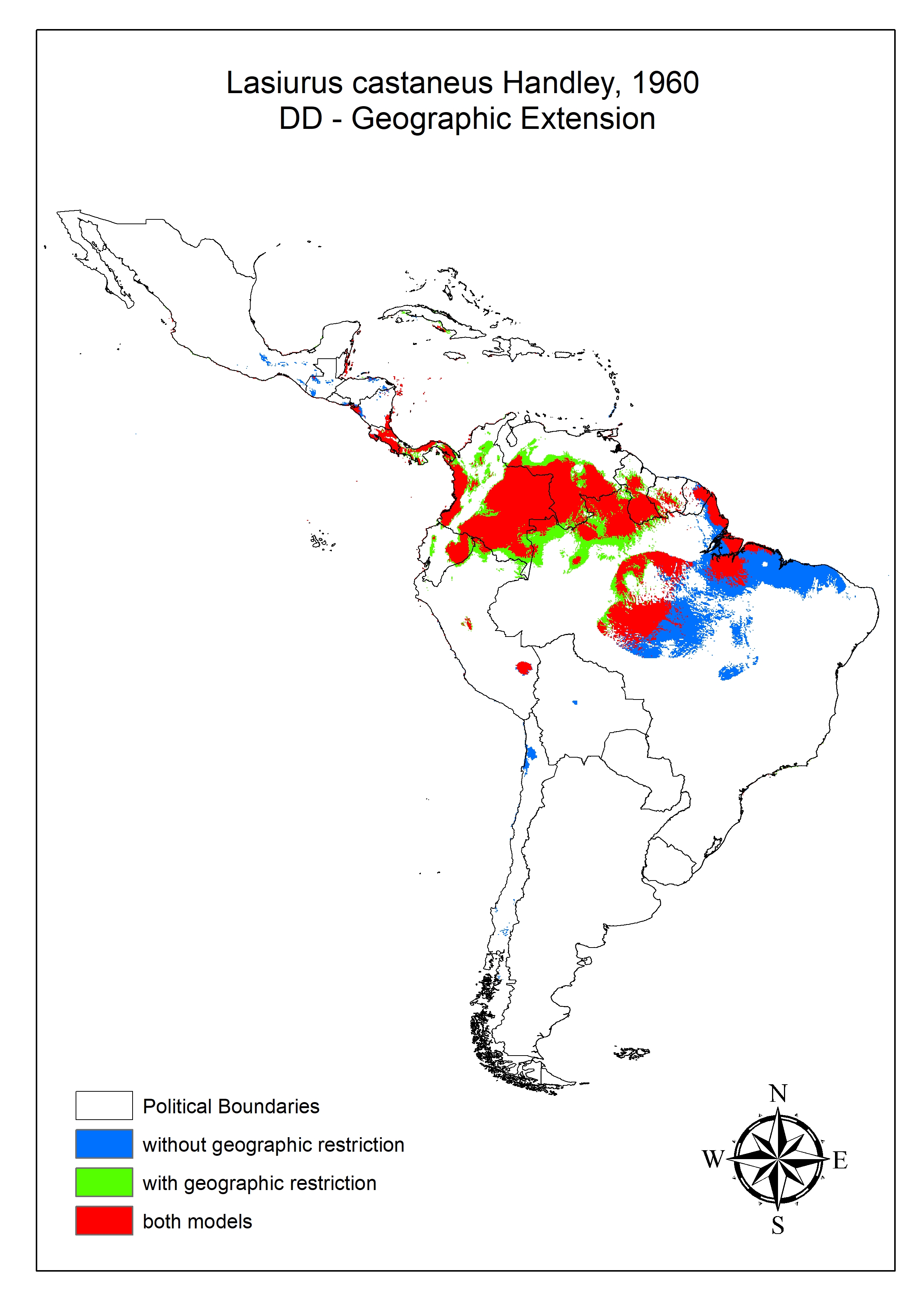

Supplement: Supplementary file 26 — Figure S24. [file ECE3-14-e11392-s006.jpg]
